# Changes in Metal Ion Concentrations in a Chardonnay Wine Related to Oxygen Exposure during Vinification

**DOI:** 10.3390/molecules24081523

**Published:** 2019-04-17

**Authors:** Marlize Z. Bekker, Martin P. Day, Paul A. Smith

**Affiliations:** The Australian Wine Research Institute, P.O. Box 197, Glen Osmond, SA 5064, Australia; martin.day@awri.com.au (M.P.D.); paul.smith@wineaustralia.com (P.A.S.)

**Keywords:** wine, metals, ICP-MS, reductive winemaking, oxidative winemaking, copper, iron, zinc, aluminium

## Abstract

The impact of oxygen exposure during winemaking on metal ion concentrations in wine were investigated throughout the winemaking process in a Chardonnay wine. The concentrations of Al, Ca, Co, Cr, Cu, Fe, K, Mg, Mn, Na, Ni, Sn, and Zn were determined using inductively coupled plasma-mass spectrometry. Oxygen exposure significantly impacted 13 metal ions at different phases of winemaking. However, only the concentrations of Cr, Cu, and Fe were impacted by early oxygen exposure during pressing, with lower Cr and Cu concentrations in wines that were aerobically pressed and lower concentrations of Fe in wines that were inertly pressed. The sequestering of Al, Cu, Ni, and Zn by wine lees was significantly affected by oxygen treatment, with lees collected from wines that were treated oxidatively sequestering significantly greater amounts of Cu and Zn and removing these metals from the wine supernatant. The metal ion that was most affected by oxygen exposure during pressing and handling was Cu, with significantly lower Cu measured in wines that were produced under oxidative conditions. It is known that elevated Cu concentrations have negative implications for wine aroma and flavour. This study demonstrated that oxygen management during winemaking significantly impacts metal ion concentrations in lees and wine, which may decrease the risk of developing taints and faults.

## 1. Introduction

The metal ion profiles of wines play an important role in modulating their sensory attributes. This is mainly as a consequence of oxidation–reduction reactions, which impact organoleptic characteristics such as wine colour, aroma, astringency and mouth feel [1,2,3,4,5,6]. Wine aroma compounds associated with unfavourable ‘reductive’ aromas are significantly impacted by residual metal ion concentrations of a wine, with a number of studies demonstrating the importance of Al, Cu, Fe and Zn in modulating the release and/or formation of compounds such as hydrogen sulfide and methanethiol [3,7,8]. These metal ions (Al, Cu, Fe, Zn) as well as metal ions such as Co, Mn, and Ni have the ability to catalyse redox reactions related to wine oxidation [9,10,11] as well as turbidity and haze formation [4,12]. Certain metal ions, such as Ca, Cu, Fe, K, Pb, and Zn, are also routinely monitored in wineries [13]. 

Furthermore, the metal ion composition of wines can be used to determine wine authenticity [6,14], which makes it critical to understand how winemaking practices may influence residual metal ion profiles in finished wine.

Factors affecting residual metal ion concentrations in finished wines include absorption from natural sources such as the vineyard soil (e.g., Al, B, Ba, Li, Mg, Mo, Si, Sr, Ti) [15], with the amount of metal ions introduced from the vineyard affected by the type of soil as well as the capacity of the grapes to absorb the various metal ions [13]. Metal ions can also be introduced into the wine through agrochemical treatments in the vineyard (e.g., Ca, Cu, Cd, K, Mn, Na, Pb, Zn) [6], environmental pollution (e.g., Cd, Co, Cr, Hg, Ni, Pb, V) [15,16], absorption from winemaking equipment (e.g., Al, Cd, Cr, Cu, Fe, Zn) [6,16], as well as through the intentional application of fining agents, such as bentonite and copper sulfate (e.g., Al, Ca, Cd, Cr, Cu, Fe, Zn) [4,6,16]. The metal ion profile of a wine may change during winemaking as a consequence of processes such as filtering, pH adjustments, yeast hull addition (e.g., Ce, Cu, Fe, La, Sb, U, V, Y) [17], and bentonite fining (e.g., Al, Ba, Be, Ca, Ce, Co, Cr, Cs, Cu, Fe, Ga, La, Li, Mg, Mn, Nd, Pr, Rb, Sb, Sr, Tl, U, V, Y, Zn) [17,18]. Bimpilas et al. [19] showed that Cu, Fe, and Mn concentrations changed significantly during the fermentation and storage of Shiraz and Merlot wines. Similarly, exogenous factors such as packaging material and storage conditions impact the metal ion profile of wines, i.e., through the use of screw caps (Sn, V), stainless steel storage vessels (Cr), as well as the storage temperature (Cu, Pb, Sn, V) [20]. As the concentrations of numerous metal ions are susceptible to changes during vinification, it is important to not include these metal ions in studies aimed at ascertaining the geographic origin of a wine. 

The modulation of metal ion concentration during winemaking processes such as pressing and handling are not as well defined as the major intervention steps described above. The binding capacity and binding affinity, metal ion oxidation state, as well as the stability of the metal ion complexes may all be subject to change as a result of varying oxygen exposure regimes; this in turn will affect the final residual concentration in wines as many metal ions may be removed through binding with grape and wine solids (lees), which ultimately leads to their removal from the wine supernatant.

The use of oxygen during winemaking is an important tool in managing fermentation efficiency, wine styles, and wine aroma [21]. Various stages of the winemaking process are impacted by oxygen. The manner in which the juice is prepared (i.e., mechanical harvesting, crushing, or whole-bunch pressing) is the first opportunity for introducing different levels of oxygen exposure and different durations of oxygen exposure, which have significant impacts on the aromatic, phenolic, and protein profiles of wine [22]. Furthermore, the prevalence of certain taints and faults, such as “reductive” aromas can be mitigated through oxidative handling of wines during fermentation [23] and oxygen management post-bottling [3,24]. A recent study showed a correlation between oxygen treatment during winemaking and the residual concentration of Cu, Fe, and Zn in a Shiraz wine [25]. Significantly lower concentrations of Cu, Fe, and Zn were measured in oxygen-treated wines compared to oxygen-deprived wines when analysed post-bottling [25]. This decrease in Cu, Fe, and Zn concentrations in the oxygenated wines may be indicative of the binding between these metal ions and wine solids, which may have removed the metal ions from the wine supernatant. In this study [25], the temporal changes in metal ion concentration as a function of oxygen exposure were not assessed, and the putative sources responsible for the removal of metal ions from the wine were also not evaluated. The effects of oxygen on the oxidation states and reactivity of metal ions as well as the available binding sites and reactivity of compounds present in the fermenting must, lees, and wine may have considerable effects on the residual metal concentration in finished wine. Understanding how oxygen exposure at different vinification stages modulates the final concentration of metal ions in grape solids post-pressing, in wine lees, as well as in finished wine will provide winemakers with tools to manage the final residual metal ion concentrations in wine. Additionally, if oxygen treatment during winemaking affects the metal ion profiles in finished wines it will also have significant impacts on wine authenticity studies. 

The aim of this study was to investigate the effect of oxygen during pressing and handling in modulating the metal ion composition in a Chardonnay wine throughout vinification, and as such determine whether certain metal ions preferentially accumulate in specific wine fractions (i.e., grape solids post-pressing, wine lees, or in the finished wine supernatant). In this study, the different levels of oxygen exposure were achieved through inert and oxidative press conditions, as well as the protected or oxidative handling of juice and just-fermented wine [22]. The metal ions investigated included macro metal ions, metal ions known to have catalytic abilities, as well as metal ions introduced into wine through metal winemaking equipment. 

## 2. Results and Discussion

### 2.1. Impacts of Oxygen Exposure during Pressing and Handling on the Metal Ion Concentration of Wine

Of all of the 22 metal ions considered for analysis, only Al, Ca, Co, Cr, Cu, Fe, K, Mg, Mn, Na, Ni, Sn, and Zn were measurable in the juice, wine, and lees samples. Metal ions such as Ag, As, Au, Cd, Hg, Pb, Pd, Pt, and Se were not detected above their limits of quantitation (LOQ) in the any of the Chardonnay samples measured in this study using inductively coupled plasma-mass spectrometry (ICP-MS) analysis and the method described by Stafilov and Karadjova [26]. The concentrations of all metal ions measured in wine supernatant in this study (Table S1) complied with the analytical requirements for the export of Australian wine (Table 1). After bottling, the concentrations of all the metal ions measured were within the average values that were previously reported for wine (Table 1) and metals such as Al, Cu, and Zn were below the maximum acceptable limits for metals in wine as outlined by The International Organisation of Vine and Wine (OIV) [27].

**Scheme 1:**
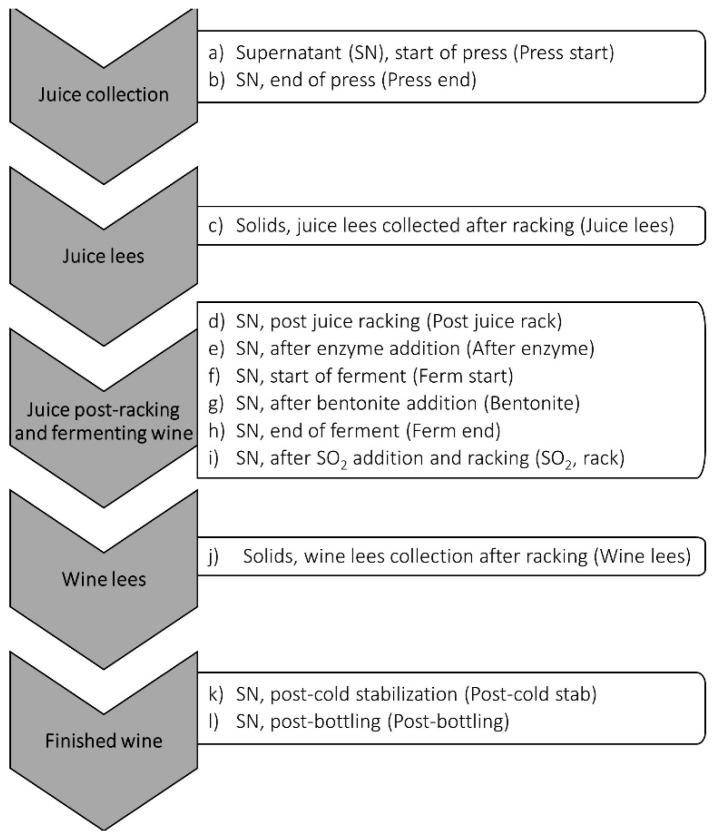
Sampling points from wine supernatant, juice lees (solids), and wine lees (solids) throughout the winemaking process. Samples were collected and the results discussed in the following sections: (1) juice collected during pressing of grapes (supernatant, SN), (a)–(b); (2) collection of grape solids/juice lees (c); (3) sample collected after juice racking to the end of fermentation (SN) (d)–(i); (4) collection of wine lees (solids), (j); and (5) samples collected from the finished wine (SN), (k)–(l).

**Figure 1:**
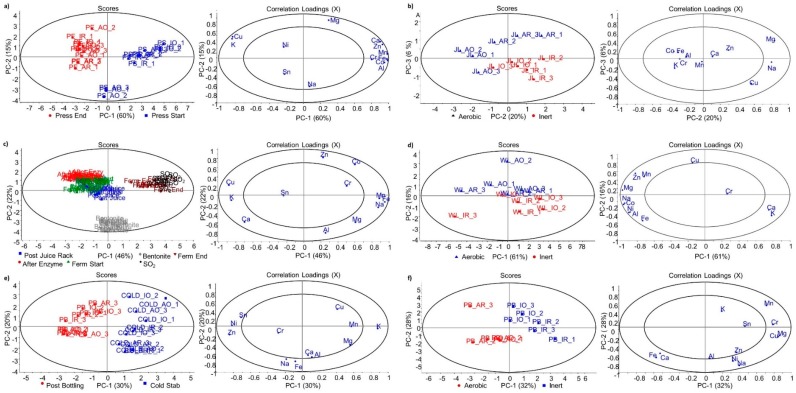
Principal component analysis (PCA) plots summarizing the differences in metal ion concentrations (*x* correlation loading plots) in Chardonnay juice, wine, and lees samples (scores plots) measured at the following time points: (**a**) juice supernatant collected at the start of press (blue) and the end of press (red); (**b**) juice lees (grape solids) collected after juice racking grouped according to the aerobic (blue) and inert pressing regime (red); (**c**) supernatant collected during fermentation, with wines grouped by samples collected after juice racking (blue), after enzyme addition (red), after the start of the ferment (green), after the end of ferment (brown), after SO_2_ addition and racking (black), and after bentonite addition (grey); (**d**) wine lees collected after racking with lees grouped by aerobically (blue) and inertly pressed (red) wines; (**e**) finished wines with samples grouped by those collected after cold stabilization (blue) and post-bottling (red); and (**f**) post-bottling wines grouped according to the aerobic (red) and inert (blue) pressing regime.

There were 12 sampling points throughout winemaking and the sampling points are discussed in five sections in Scheme 1, namely: juice, (a)–(b); juice lees, (c); fermentation, (d)–(i); wine lees, (j); and finished wine, (k)–(l). The effects of vinification and specifically oxygen treatment during pressing and handling on these five stages of the winemaking process are discussed below, and the results are summarised in six principal component analysis (PCA) plots that provide an overview for the results and discussion sections (Figure 1a–f). 

Metal ion concentrations are expressed in mg/Kg and comparisons are made within the wine supernatant samples and grape and wine solid samples. Statistical analysis methods (two-way ANOVA) were used to compare the significant increases and decreases in metal ion concentrations associated with oxygen exposure in wine supernatant samples as well as in the solid samples. 

#### 2.1.1. Metal Ion Composition of Grape Juice 

Juice samples were collected directly from the pressing vessel as the first juice was pumped into the press holding tank [Press start, Scheme 1 (a)] and again at the end of pressing [Press end, Scheme 1 (b)]. At the start of the press, the juice contained significantly larger concentrations of Al (*p* value < 0.0001), Ca (*p* value 0.0148), Co (*p* value < 0.0001), Cr (*p* value 0.0048), Fe (*p* value < 0.0001), Mn (*p* value 0.0002), and Zn (*p* value 0.0061) compared to the juice measured at the end of press (Figure 1a). It is known that metal ion concentrations may vary during vinification [6], with several reasons for variation in metal ion concentrations during this phase of winemaking. Increased concentrations of Cu and K at the end of press compared to those measured in juice at the start of press may occur as the result of increased extraction from the naturally occurring metal ions obtained from grape berry (K) or artificial sources, such as from residual pesticides, fungicides, and/or fertilizers introduced from the grape skins during crushing (Cu) [5,6]. Initially, the Cu and K concentrations may appear lower as all the elements have not yet been completely extracted from the grape solids at the start of press, whereas at the end of pressing, the metals had time to more fully extract from the grape solid material. The rate of extraction from the natural or artificial sources may also vary, with the highest concentration of certain metal ions extracted in the first aliquot at the start of press, with less metal ions remaining for extraction at the end, which may have resulted in decreased Al, Ca, Co, Cr, Fe, Mn, and Zn concentrations when comparing the samples collected at the start and end of pressing. Another factor to consider is that the metal ion concentrations of Al, Ca, Co, Cr, Cu, Fe, K, Mn, and Zn may also decrease as insoluble tartrate precipitates are produced in the juice and must, which would remove the metal ions from the supernatant, as discussed by Pohl 2007 [6] and Galani-Nikolakaki et al. [30].

**Figure 2:**
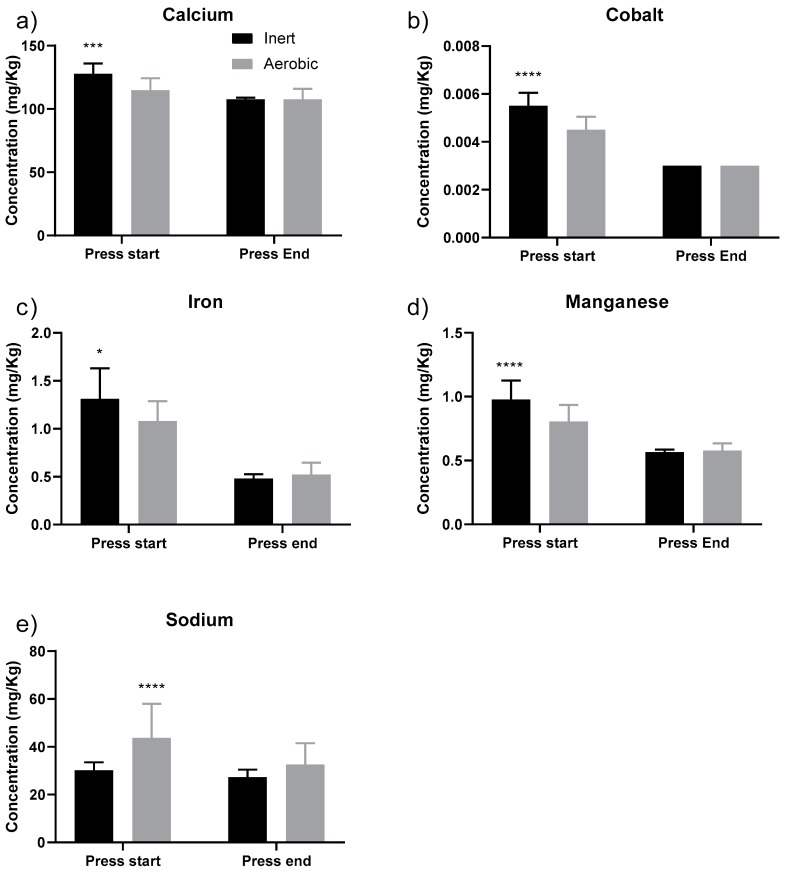
Ca (**a**), Co (**b**), Fe (**c**), Mg (**d**), and Na (**e**) metal ions measured in Chardonnay juice that were significantly impacted by oxygen exposure during pressing, with juices obtained using an inert pressing technique (“Inert”, black) compared to juices obtained using an aerobic pressing technique (“Aerobic”, grey). Significant interactions are indicated as follows: not significant (ns) for *p* value > 0.05; * *p* value ≤ 0.05; ** *p* value ≤ 0.01; *** *p* value ≤ 0.001; **** *p* value ≤ 0.0001.

When considering only the effect of pressing conditions (inert vs. aerobic) on metal concentrations in the juice, during this early stage of vinification, aerobic pressing conditions impacted Ca, Co, Fe, Mn, and Na concentrations (Table S4a,b), with all the metal ions except Na occurring in larger concentrations in inertly pressed juice compared to aerobically pressed juice (Figure 2a–e). The lower concentrations of Ca, Co, Fe, and Mn in the aerobically pressed juices may be explained by the complexation of these metal ions with oxidation reaction products produced from the solid grape material and the fact that this reaction proceeded faster under aerobic conditions. Another possibility, as discussed previously, is that exposure to oxygen increased the formation of insoluble metal–tartrate complexes [6,22], which resulted in the faster removal of these metal ions from the collected juice at the start of press, but that the total amount of metal ions removed from aerobically pressed and inert samples were comparable when measured again at the end of pressing (Figure 2a–e).

At the end of the press cycle, no differences were measured in the inertly pressed juices compared to the aerobically pressed juices (Figure 2). This suggests that the initial differences were transient and a reflection of the timing of sample collection rather than the impact of the aerobic or inert pressing conditions.

#### 2.1.2. Metal Ion Concentrations in Juice Lees

**Scheme 2:**
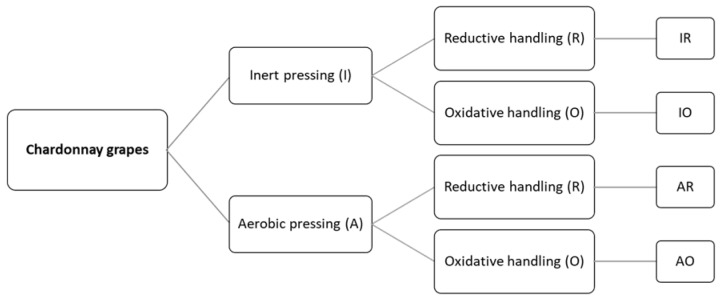
Experimental design showing the four combinations of oxygen exposure regimes during pressing and handling: inert-reductive (IR), inert-oxidative (IO), aerobic-reductive (AR) and aerobic-oxidative (AO).

After the inert/aerobic pressing of the grape berries [Scheme 1 (a)–(b)], the juice as well as the wines were handled either reductively or oxidatively for the duration of vinification (Scheme 2), as described by Day et al. [22]. This resulted in a gradient of oxygen exposure that increased in the following order: inert pressing–reductive handling (IR) < inert pressing–oxidative handling (IO) < aerobic pressing–reductive handling (AR) < aerobic pressing–oxidative handling (AO).

The major differences in the metal ion concentrations of juice lees collected from juices that were pressed either aerobically or inertly were in Cu, Mg, and Na concentrations, with higher concentrations of these metal ions in juices that were pressed inertly compared to juices that were pressed aerobically (Figure 1b). 

**Figure 3:**
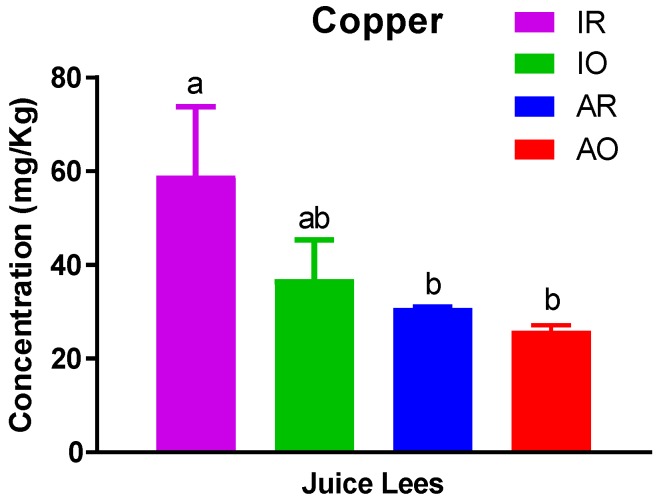
Effects of oxygen exposure during pressing and handling on the concentration of Cu in Chardonnay juice lees for the treatments inert pressing–reductive handling (IR, purple), inert pressing–oxidative handling (IO, green), aerobic pressing–reductive handling (AR, blue), and aerobic pressing–oxidative handling (AO, red). Significant differences between treatments are indicated by different letters above each bar graph as determined by ANOVA.

Only Cu was significantly affected by oxygen exposure during pressing and handling, with lower Cu concentrations measured in grape solids exposed to the largest amount of oxygen (Figure 3, Table S5a,b). Cu may be introduced through extraction from the grape berry, from contamination of Cu-containing vineyard treatments remaining on the grape skins post-treatment. The lower concentration of Cu present in wines treated oxidatively suggests that either (1) the binding of Cu to grape solids and its subsequent removal from the wine was increased by inert pressing, or (2) oxygen exposure increased the ability of Cu to bind to soluble wine compounds such as anthocyanins, polyphenols, and proteins, thus it was retained in the wine matrix and was not measured in the grape solids/juice lees [31].

Interestingly, the significant effects of oxygen exposure during pressing on the metal ion concentrations in juice were not reflected in the concentrations of Ca, Cr, Fe, Mn, or Na in juice lees (Tables S5 and S6). This suggests that the sequestering of these metal ions by the grape solids was not affected by inert or aerobic pressing and oxygen exposure, or the lack thereof, did not significantly impact the ability of these metal ions to bind to grape cell wall material, grape proteins, or other compounds present in the juice lees.

#### 2.1.3. Metal Ion Concentrations in Fermenting Wine

Twelve metal ions (Al, Ca, Co, Cr, Cu, Fe, K, Mg, Mn, Na, Sn, Zn) were measured in the fermenting wine during this stage of vinification. All twelve metal ions were significantly affected at some point during this stage of vinification through different oxygen exposure regimes from post-racking from the juice solids to before the removal of the wine lees [Scheme 1 (d)–(i), Tables S3 and S4]. 

**Figure 4:**
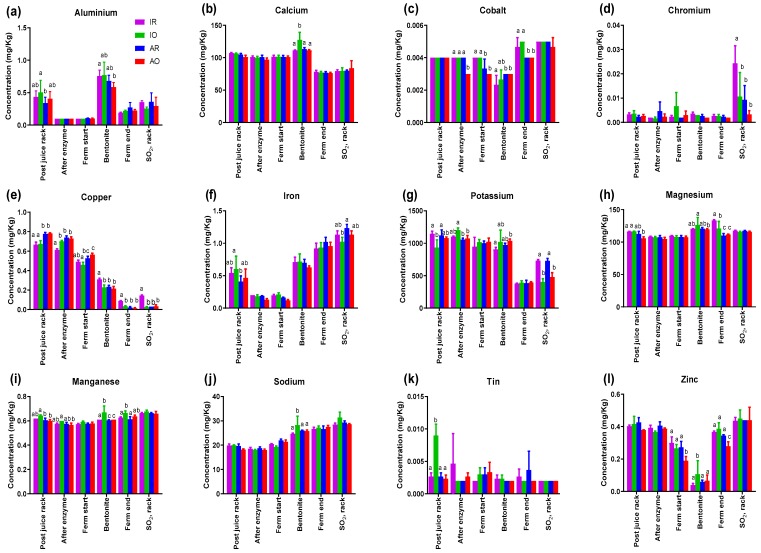
Effects of oxygen exposure during pressing and handling on the concentration of Al (**a**), Ca (**b**), Co (**c**), Cr (**d**), Cu (**e**), Fe (**f**), K (**g**), Mg (**h**), Mn (**i**), Na (**j**), Sn (**k**) and Zn (**l**) in Chardonnay wines during the fermenting stage for inert pressing–reductive handling (IR, purple), inert pressing–oxidative handling (IO, green), aerobic pressing–reductive handling (AR, blue), and aerobic pressing–oxidative handling (AO, red) treatments. Significant differences between treatments are indicated by different letters above each bar graph as determined by ANOVA.

The fermenting wine was separated into three unique groups according to its metal ion concentrations, namely the early stages of vinification (“Post juice rack”, “After enzyme”, “Ferm start”), the samples collected after bentonite addition (“Bentonite”), and the samples collected during the final stages of fermentation (“Ferm end”, “SO_2_”) (Figure 1c). During the start of vinification (“Post juice rack”, “After enzyme”, “Ferm start”), the samples contained larger concentrations of Ca, Cu, and K than the other wine samples, and the samples collected after bentonite addition (“Bentonite”) contained larger concentrations of Al than the other samples (Figure 1c). Towards the end of fermentation (“Ferm end”, “SO_2_”), the wines contained higher concentrations of Al, Co, Cr, Fe, Mg, Mn, Na, Zn than the samples collected at the start of vinification (“Post juice rack”, “After enzyme”, “Ferm Start”) (Figure 1c). Overall, the concentrations of the majority of the metal ions (Ca, Co, Cr, K, Mg, Mn, Na) remained relatively stable up to bentonite addition (Figure 4). Only Al, Cu, Fe, and Zn decreased in concentrations after juice racking (“Post juice rack”) and before bentonite addition, which suggests that these metal ions were sequestered by the lees and other wine compounds present in the fermenting wine. The solid material then settled to the bottom of the fermenting tank and the metal ions bound to the solids were systematically removed from the supernatant. Cu showed a stepwise decrease in concentration as vinification progressed (Figure 4). It is known that Cu binds strongly to lees [32] and its complexation with other wine matrix compounds are to be expected. Zinc also significantly decreased in concentration as the fermentation stage neared its end, suggesting increased binding of Zn to the lees. 

Bentonite fining is a major winemaking intervention and is known to contribute to increased metal ion concentrations in wine, as previously described by Catarino et al. (2008) [33]. Rossano et al. (2007) showed that the concentrations of many transition metals and rare earth elements used for authenticity studies also significantly change after bentonite treatment [34], which will negatively impact authenticity investigations as the relationship between wine and soil elemental composition may be compromised [35]. The addition of bentonite during active fermentation is more efficient in achieving protein stability, and it also increases the fermentation rate and has less of an impact on the sensory properties of wines [36]. There were significant increases in the average concentrations Al, Ca, Fe, Mg, and Na measured in all wines immediately after bentonite addition [Scheme 1 (g)]. The Al concentrations increased by 583% (*p* value > 0.0001), Ca concentrations increased by 14% (*p* value 0.0187), Fe concentrations increased by 300% (*p* value > 0.0001), Mg concentrations increased by 12% (*p* value 0.0039), and Na increased by 26% (*p* value 0.0002) (Figure 4a,b,f,h,j), which is in agreement with numerous previous studies that reported increased Al, Ca, Fe, and Mg concentrations, among others [4,6,16,17,18]. Only Fe and Na concentrations remained elevated post-bentonite addition and throughout the rest of the winemaking process [Scheme 1 (g) to (i), (k) to (l)], with Fe and Na concentrations increasing on average by 592% and 68%, respectively (Figure 4f,j, Table S1). Co, Cu, and Zn concentrations decreased significantly in wine samples measured immediately after bentonite treatment, with Co decreasing by 23% (*p* value > 0.0001), Cu decreasing by 52% (*p* value > 0.0001), and Zn decreasing by 73% (*p* value > 0.0001), however, only Cu remained lowered for the rest of vinification (Figure 4e).

During the final stages of fermentation [Scheme 1 (h), (i)] Al, Co, Mg, Mn, and Zn concentrations returned to approximately the same concentrations as their post-juice racking concentrations (Figure 4a,c,h,i,l). For Al and Mg, significantly increased concentrations were measured directly after bentonite treatment; however, after the wines were racked off the bentonite lees, the concentration of these metal ions decreased to approximately the same concentration as before the treatment (Figure 4a,h), which suggests that the metal ions introduced during bentonite fining were sequestered by the yeast or other solid material during active fermentation, or that tartrate complexes of Al and Mg were produced that settled out of the wine supernatant. Ca concentrations significantly increased (14%, *p* value 0.0187) when measured immediately post-bentonite fining in all wine samples, however, this was followed by decreased concentrations (Figure 4b), most likely due to the formation of calcium tartrate precipitates that settled to the bottom of the fermentation vessel and as such systematically removed Ca from the wine supernatant. As mentioned earlier, bentonite treatment was associated with significantly decreased Co, Cu, and Zn; however, given that bentonite was added to the active ferment in this study, the bentonite was exposed to the wine supernatant for a longer period than if it was added just before SO_2_ addition at the end of ferment. This allowed time for the dissociation of Co and Zn from the bentonite or dissociation from other Co- or Zn-complexes and thus the concentrations of Co and Zn returned to approximately the same levels as pre-bentonite fining (Figure 4c,l). The concentrations of Cu in all of the wine samples continued to decrease during vinification (Figure 4e), which is in agreement with previous studies by Rousseva et al. 2016 [37] and Castineira Gomez et al., 2004 [18], which showed that Cu concentration decreased following bentonite treatment and that the major loss occurred from the cationic fraction of Cu [37].

The addition of SO_2_ had a major effect on the Cr concentrations (Figure 4d), with significantly increased Cr concentrations (*p* value 0.0225) in all wines following SO_2_ addition. This suggests that the addition of SO_2_ facilitated the extraction of Cr from the steel alloy fermentation vessels. In contrast, the concentration of Fe continued to increase while the wines were exposed to bentonite [pre-wine lees removal, Scheme 1 (j)], which suggests that Fe was continuously extracted from the bentonite. This is in agreement with previous studies by Rousseva et al., 2016 [37] and Castineira Gomez et al., 2004 [18], which showed that bentonite treatment increased Fe concentration, and interestingly, that it was mainly the cationic fraction of Fe that increased after bentonite treatment [37]. The concentrations of K were unaffected by bentonite treatment; however, at the end of fermentation, K concentrations decreased significantly by 44% on average when measured in all samples post-bentonite addition (Figure 4g). This suggests that potassium tartrate was produced and that it settled to the bottom of the fermentation vessel, removing K from the fermenting wine supernatant. 

Oxygen exposure during pressing and vinification significantly impacted all measured metal ions at certain stages of vinification; however, many of these effects were only measured at one timepoint and did not have significant long-term impacts on the metal ion concentrations in wines at the end of fermentation (Figure 4, Tables S3 and S4). For example, oxygen exposure significantly affected K concentrations; however, the effects were not consistent from timepoint to timepoint (Figure 4g). Similarly, Co appeared to be significantly impacted by oxygen exposure, with oxygen exposure playing a significant role in modulating Co concentrations in grape juice (Section 2.1.1, Figure 2b) as well as in fermenting wines (Figure 4c). However, after SO_2_ addition and racking of the lees, there were no significant differences in Co concentrations between treatments (Figure 4c). The impact of oxygen exposure on the amount of Cr that was introduced into the fermenting wines after SO_2_ addition was significant, with significantly greater Cr concentrations measured in wines that were completely protected from oxygen (IR) and the lowest concentration of Cr measured in wines that were exposed to the largest amount of oxygen during vinification (AO) (Figure 4d). Hopfer et al. (2015) have shown that metal ions such as Cr and Fe (among others) show significant differences related to the winery equipment used [16] and this current study has highlighted that the use of SO_2_ may further impact the ability of Cr to leach from metal alloy winery equipment.

Of all the metal ions evaluated in this study, Cu was the most affected by oxygen treatment and the effects of oxygen exposure on Cu concentrations can be followed from the impacts that oxygen had on the concentrations of Cu in the juice lees (Section 2.1.1) and all throughout fermentation. As discussed in Section 2.1.1, the juice lees collected from reductively treated samples contained higher concentrations of Cu (Figure 3). Given that a larger portion of Cu was removed from the inertly pressed samples when the juice lees were removed, a relatively higher concentration of Cu in the aerobically pressed juices was expected, as a larger amount of Cu was removed from the inertly pressed samples after juice racking. The relatively higher Cu concentrations associated with oxygen-exposed wines were maintained up to the addition of bentonite (Figure 4e). However, following bentonite treatment, all wines exposed to oxygen at any stage of vinification (IO, AR, AO) contained significantly lower Cu concentrations than the wines that were completely protected against oxygen exposure (IR) (Figure 4e). This effect is most likely due to the complexation and binding of Cu to either bentonite or insoluble wine matrix compounds during active ferment, which slowly settled to the bottom of the fermenting vessel. As such, Cu was removed from the wine supernatant. These results suggest that the binding capacity of Cu to insoluble wine matrix compounds increased in samples exposed to oxygen. The exact causes for this effect are unclear, but it may be related to the oxidation state of Cu(I) vs. Cu(II) or the formation of oxidation grape or yeast reaction products that modulated the scavenging ability of the solids in the wine as well as the ability of Cu to bind with bentonite. 

Interestingly, for certain metal ions (Al, Ca, Co, Cu, K, Mn, and Na) it appears that oxygen exposure significantly affected the impact of bentonite addition on their concentrations in wine. When measured directly post-bentonite addition, wines exposed to oxygen contained significantly higher concentrations of Co, Cu, and K, and significantly lower concentrations of Al (Figure 4a,c,e,g, Table S4a,b). Significantly higher Ca concentrations were measured in IO-treated wines; however, this significant difference was only measured directly post-bentonite addition. This suggests that the oxygen-exposed wines extracted higher concentrations of Co, Cu, and K from bentonite.

#### 2.1.4. Effects of Oxygen Exposure on Metal Ion Concentrations of Wine Lees

The differences in metal ion concentrations of the lees collected after SO_2_ addition and after the wines were cold-stabilised are mainly evident in Cu concentrations (Figure 1d, Tables S5 and S6). The biosorption mechanisms of metals by *Saccharomyces cerevisiae* are complicated and not fully understood [32]; however, certain mechanisms have been elucidated, including the sequestering of copper by lees proceeds through binding on the yeast cell wall, with functional groups such as amino, carboxyl, phosphate, phosphodiester, and hydroxyl groups known to play important roles in the complexation of heavy metal ions [32,38,39]. Copper can also accumulate in yeast through the formation of inclusion bodies or by binding to yeast proteins [32]. 

**Figure 5:**
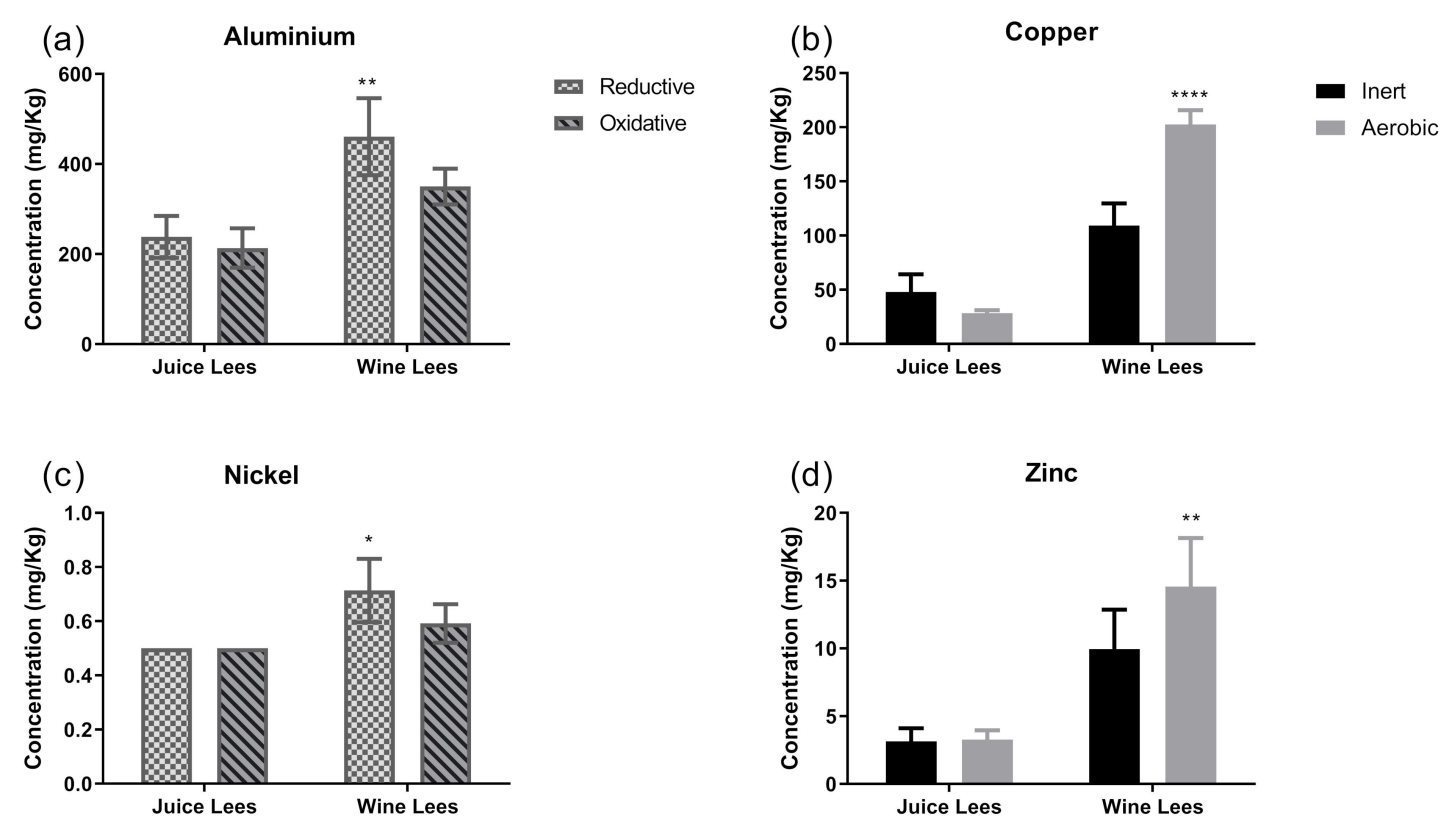
Effects of oxygen exposure during pressing and handling on the concentration of Al (**a**), Cu (**b**), Ni (**c**), and Zn (**d**) in Chardonnay juice lees and wine lees for inert pressing (“Inert”, black), aerobic pressing (“Aerobic”, grey), reductive handling (“Reductive”, chequered grey), and oxidative handling (“Oxidative”, striped grey) treatments. For *p* value > 0.05; * *p* value ≤ 0.05; ** *p* value ≤ 0.01; *** *p* value ≤ 0.001; **** *p* value ≤ 0.0001.

Only Al, Cu, Ni, and Zn concentrations were significantly impacted by oxygen exposure during vinification. Lees collected from wines exposed to oxygen during handling and processing contained significantly lower Al and Ni concentrations (Figure 5a,c, Table S6a,b) compared to lees collected from wines that were protected from oxygen exposure. For both Cu and Zn, it was the oxygen exposure during grape pressing that determined the concentrations of these two metal ions in the wine lees, and for both of these metal ions, lees collected from wines produced from aerobically pressed grapes (“Aerobic”) contained significantly higher concentrations than lees collected from wines produced from inertly pressed grapes (“Inert”) (Figure 5b,d, Table S6a,b). The type of reaction products produced in the juice during aerobic pressing conditions may to be key in modulating the binding capacity of the lees post-fermentation. These compounds may include quinones, polyphenols, tannins, aldehydes, glutathione, proteins, and other wine compounds that are affected by oxygen exposure.

#### 2.1.5. Effects of Oxygen Exposure on Metal Ion Concentrations of Finished Wine

Evaluating the metal ion concentrations in the wine during the final phases of winemaking and post-bottling [Scheme 1 (k)–(l)] is key to determining the relevance of the effects discussed in the previous sections. The effect of oxygen treatment during the earlier phases of winemaking is not of great importance if those effects are not observed in bottled wine. The main differences between samples taken from wines after cold stability and those taken from wines immediately post-bottling are higher concentrations of K in the wines measured before cold stability and higher concentrations of Ni, Sn, and Zn in the wines measured post-bottling (Figure 1f). The lower concentration of K in the wines measured post-bottling could be explained by the removal of potassium tartrate precipitates during filtration, which is a standard procedure that takes place before bottling. Both Ni and Sn are commonly used to produce screw caps and the increase in these metals in wines measured post-bottling may be related to leaching of these metal ions from the screw caps, as previously described by Hopfer et al. (2013) [20]. It is important to note that although an increase in the concentrations of these metal ions was measured, the concentration of Ni in the finished wines was lower than the World Health Organisation (WHO) recommendation for safe levels of Ni in water, which is 70 µg/L [40]. Due to the low absorption of Sn and inorganic Sn compounds by the gastrointestinal tract, Sn is rapidly excreted and does not pose a hazard to human health; therefore, the WHO has not established a guideline for the value for inorganic Sn [40]. However, the maximum concentration of Sn in these wines was considerably low at 9 µg/Kg.

**Figure 6:**
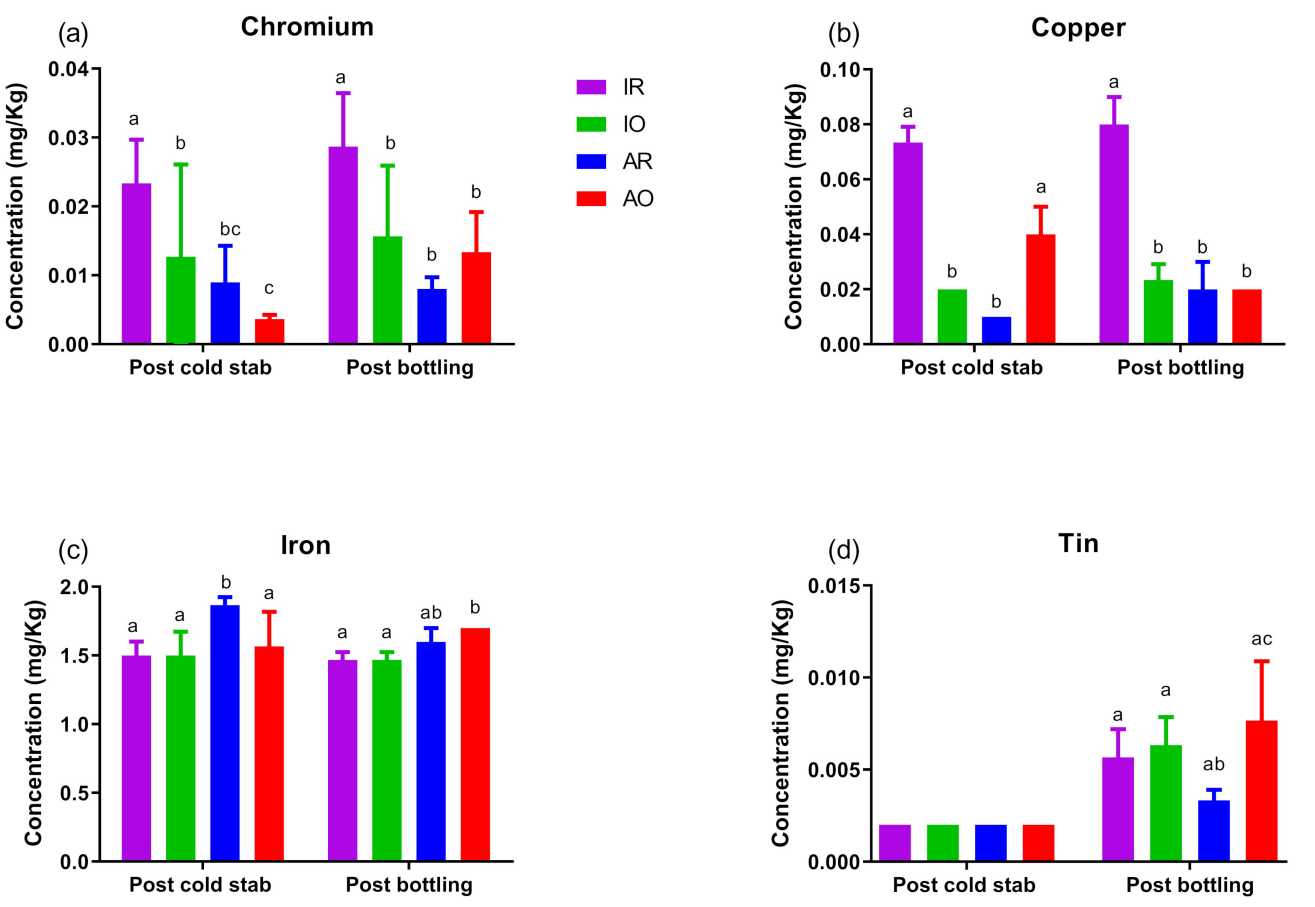
Effects of oxygen exposure during pressing and handling on the concentration of Cr (**a**), Cu (**b**), Fe (**c**) and Sn (**d**) in finished Chardonnay wines for inert pressing–reductive handling (IR, purple), inert pressing–oxidative handling (IO, green), aerobic pressing–reductive handling (AR, blue), and aerobic pressing–oxidative handling (AO, red) treatments. Significant differences between treatments are indicated by different letters above each bar graph as determined by ANOVA.

Oxygen exposure during pressing and handling had significant long-term effects only on Cr, Cu, and Fe. The concentration gradient of Cr in wines that related to the amount of oxygen that the wines were exposed to during SO_2_ addition (reductive > oxidative) was still apparent in the wines after cold settling as well as post-bottling (Figure 6a). Cr was not removed when the wines were racked, which suggests that Cr does not bind as readily to yeast lees as metal ions such as Cu. The wine lees was effective in binding Cu and wines exposed to the largest amount of oxygen during winemaking bound the largest amount of Cu (Figure 5b); the Cu was removed when the wines were racked, producing wines with the lowest Cu concentrations (Figure 6b). In this study, aerobic pressing of juice had the most significant effect on final Cu concentrations; however, even if grapes were pressed inertly, significantly lower Cu concentrations were measured in the wines that were handled oxidatively in the winery during vinification (Figure 6b). 

As previously mentioned, Cu was the metal ion most affected by oxygen exposure during pressing and handling, and significantly lower Cu concentrations were achieved in wines by handling the wines oxidatively during vinification. It is known that Cu negatively impacts the organoleptic properties of wine, which includes modulating the formation of hydrogen sulfide, a compound associated with “reductive” aromas in wine. Recent studies have shown that Cu is fundamental in the formation of putative precursors to hydrogen sulfide [9,41], and these precursors have the potential to later release hydrogen sulfide post-bottling [8]. It is known that wines produced under oxidative conditions have lower concentrations of compounds associated with “reductive” aromas [23]. This study supports the findings of the above-mentioned studies by demonstrating that wines produced under oxidative conditions contain lower concentrations of metal ions such as Cu due to the removal of Cu through its sequestration by lees in wines that were handled oxidatively. The Cu may be bound in such a manner that prevents it from participating in formation reactions of precursor compounds. This is followed by its removal from the supernatant after racking, which prevents it from participating in “reductive” aroma compound release reactions in wines post-bottling.

Oxygen exposure during pressing greatly impacted Cr concentrations. The lowest concentration of Cr was found in wines produced from aerobically pressed grapes, with concentrations decreasing in the following order: IR > IO ~ AR > AO (Figure 6a). It appears that the manner in which grapes are pressed (aerobic vs. inert) plays a significant role in determining the final Cr profiles in fermenting wines. This may be related to the formation of oxidized grape compounds with the capacity to bind Cr later during fermentation, or the oxidized grape compounds or higher concentration of oxygen may preferentially react with the added SO_2_ and protect the fermentation vessel from the effects of SO_2_, thus limiting the extraction of Cr into the fermenting wine from the steel alloy fermentation vessel. Fe continued to increase during winemaking and was most likely introduced through the winemaking equipment. The increase in Fe concentrations plateaued after SO_2_ and racking and remained constant to the post-bottling phase (Figure 4f and Figure 6c). There was a small oxygen effect on Fe concentrations, and the data suggest that oxygen exposure during pressing was important in determining the final Fe concentrations in these wines (Table S4a). The role of both Cu and Fe in wine oxidation is well known [42], with the detrimental effects of increased residual Cu concentration on protein haze formation as well as sensory qualities also being well-defined [43]. Oxygen exposure during vinification also had significant effects on the concentration of Sn in the wines post-bottling. This was the only point were variation in Sn concentrations was measured. The aerobically pressed wines that were handled aerobically (AO) contained higher Sn concentrations than the wines handled reductively (AR), which may indicate that exposing wines to higher amounts of oxygen during winemaking may influence the ability of Sn to be introduced from the liner of the screwcap closure. This is in agreement with results from Hopfer et al. [20] who showed that wines with a lower fill height and stored at a higher temperature had higher Sn concentrations. For our samples, all of the wines were stored at the same 15 °C; however, the lower fill height in the samples of the Hopfer et al. study would have created a more oxidative environment, which is comparable to our oxygen-exposed wines (AO).

In conclusion, in this study, different levels of oxygen were applied through normal pressing and handling operations. The choice of aerobic or inert pressing mode as well as the extent to which the fermenting wine was exposed to or protected from oxygen during handling were both shown to significantly impact the final residual metal ion profiles of the Chardonnay wines. Twelve metal ions were significantly affected by oxygen exposure at different phases of winemaking; however, only the residual concentrations of Cr, Cu, Fe, and Sn in the bottled wine were impacted by oxygen exposure during pressing and handling. Of all the metal ions evaluated in this study, Cu was the most significantly impacted by oxygen exposure throughout the winemaking process. Importantly, oxygen exposure significantly impacted the amount of Cu bound and subsequently removed by juice solids and wine lees, and this was reflected in the Cu concentration in wines post-bottling. The effects of oxygen exposure on residual metal ion concentrations in wine have important implications for the organoleptic qualities of wine as well as for wine authenticity studies, given that the metal ions most affected by oxygen exposure are known to be important for aroma development and wine oxidation. Previous studies have shown that oxygen exposure modulates the formation of “reductive” aroma compounds [23]. Considering that Cu is known to be directly involved in modulating “reductive” aromas, this study provides a putative mechanism for the lower prevalence of ‘reduced’ characters in wines produced oxidatively by demonstrating that significantly less residual Cu remains in wines exposed to oxygen during vinification. This decreases the risk of Cu being involved in redox reactions in these wines. In this study, only Mg and Mn remained relatively consistent throughout the winemaking process and were unaffected by normal winemaking interventions (such as bentonite fining, for example) and oxygen exposure during pressing or handling. Additionally, there was no absorption of Mn or Mg from winemaking equipment, and neither of these metals were sequestered by the juice solids or the wine lees. 

In summary, this study provides further evidence of the effectiveness of utilising oxidative winemaking techniques to improve wine aroma and flavour profiles, as lower concentrations of metal ions such as Cu, Al, and Zn were measured in wines exposed to oxygen during vinification than in wines that were protected from oxygen. It is known that these metal ions are associated with the formation of certain taints and faults, and as such, limiting the concentrations of these metal ions in post-bottling wines is an important consideration for winemakers. This work provides a strong basis for future studies that should aim to identify the Cu, Al, and Zn species produced during oxidative winemaking practices.

## 3. Materials and Methods 

### 3.1. Winemaking and Oxygen Exposure Regime

The wines with four different oxygen exposure regimes (IR, IO, AR, AO) were prepared as described by Day et al. [22]. Briefly, Chardonnay grapes from the Barossa region (2014 vintage) were hand-picked and whole-bunch pressed (3 tonnes per load) using a Bucher-Vaslin XPF50 Inertys^®^ (Chalonnes-sur-Loire, France). Two pressing protocols were followed, inert mode and aerobic (or normal) mode. During inert mode, two aliquots (1700 L per aliquot) of juice were pressed while the membrane press was configured to draw in nitrogen gas rather than air as the membrane deflated prior to crushing. The press was sparged with CO_2_ gas prior to and during loading. During aerobic mode, two aliquots (1700 L per aliquot) of juice were pressed while ambient air was drawn in as the press membrane deflated. The inertly pressed or aerobically pressed juice samples were subdivided into juice samples that were to be handled either “reductively” or “oxidatively” during winemaking and these samples were transferred into 500 L temperature-controlled tanks. All juice samples received a standard addition of 35 mg/L sulfur dioxide (SO_2_) in the press holding tank before transportation to the Hickinbotham–Roseworthy Wine Science laboratory at the University of Adelaide, where the inert and aerative juice treatments were cold settled. The wines were handled “reductively” by creating an inert atmosphere in the source and receival tanks (blanketing with inert gas or dry ice) during transfer and racking operations, resulting in four combinations of pressing and handling: inert pressing–reductive handling (IR), inert pressing–oxidative handling (IO), aerobic pressing–reductive handling (AR), and aerobic pressing–oxidative handling (AO) (Scheme 2). All treatments were conducted in triplicate. Bentonite was added to all samples during active ferment when the total soluble solids dropped by 1 Bé to give a concentration of 1 g/L. The reductive handling technique was continued until after post-fermentation racking, at which point 60 mg/L SO_2_ was added. The tank headspace was regularly sparged with inert gas to maintain a headspace oxygen concentration below 0.5% air saturation. For the oxidative handling, the same processes were followed as for the reductively handled wines; however, no inert gas was used during the winemaking process until after post-fermentation racking. After post-ferment racking, all four treatments were treated identically, with all subsequent winemaking operations carried out in a standard reductive manner using inert gas cover to minimize oxidation [22]. 

### 3.2. Materials and Reagents

Nitric acid (HNO_3_, 69%) was purchased from Merck (Bayswater, Victoria, Australia). Fermenting juice and wine were sampled using 10 mL pipettes (Greiner CELLSTAR^®^ serological pipettes) obtained from Sigma-Aldrich (Castle Hill, NSW, Australia) that were pre-washed with 5% HNO_3_ and storing the pipettes overnight in 5% HNO_3_ to remove all traces of residual metal ions on the pipettes’ surface. Following the acid wash the pipettes were rinsed five times using water obtained from a Milli-Q purification system (Millipore, North Ryde, NSW, Australia). Samples were stored in 15 mL screw cap digestion tubes containing lids that are certified trace metal free (DKSH, Hallam, Victoria, Australia) at −20 °C until later analysis using ICP-MS).

### 3.3. Sample Collection 

Samples (10 mL) were collected using acid-rinsed pipettes free of trace metal contaminants at each major winemaking intervention step (Scheme 1). Samples were centrifuged (10 min at 1730× *g*) in a pre-tared container, the was supernatant decanted from the solid pellet into certified trace metal-free tubes (DKSH, Hallam, Victoria, Australia), and the supernatant was stored at −20 °C until ICP-MS analysis. The solid samples were resuspended in water (Milli-Q purification system, Millipore, North Ryde, NSW, Australia), frozen at −80 °C, and lyophilized to complete dryness.

### 3.4. Analysis

#### 3.4.1. Dissolved Oxygen Measurement

Dissolved oxygen (DO) was measured inside the press using a miniDOT data logger (Precision Measurement Engineering, Vista, CA, USA) temporarily fixed near to the press’s juice channels. The mean DO concentration inside the press was 3.3% (± 2.1) air saturation (~370 ± 230 ppb) during inert pressing and 80% air saturation (7.25 ppm) during aerobic pressing [22].

#### 3.4.2. Metal Ion Analyses

Wines were analysed for the following metal ion concentrations: Ag, Al, As, Au, Ca, Cd, Co, Cr, Cu, Fe, Hg, K, Mg, Mn, Na, Ni, Pb, Pd, Pt, Se, Sn, and Zn by Flinders Analytical, Flinders University (Adelaide, Australia) using an Agilent 7500 cx inductively coupled plasma mass spectrometer (Agilent Technologies, Tokyo, Japan) as described by Stafilov and Karadjova [26] and Wheal et al. [44], with slight modification. Briefly, 20–50 mg of a solid sample (grape solids/wine lees) or a 2.5 mL juice or wine sample was acid-digested by adding HNO_3_ (4 mL) to the sample.The sample was then re-sealed and heated for 1.5 h at 100 °C in aluminium heater blocks at atmospheric pressure, after which peroxide (H_2_O_2_, 2 mL) was added and the sample heated for another 2 h at 100 °C. The sample was then made up to 50 mL. Samples were analysed as described by Stafilov and Karadjova [26], and the metal ion concentrations are given as mg/Kg sample. 

The residual concentrations for the metal ions in the juice lees/grape solids and wine lees samples were corrected by subtracting the concentration of the metal ions present in the supernatant of the wet lees from concentrations of the metal ions determined in the dry lees. 

The average and standard deviation (Stdev) for the concentrations (mg/Kg) of the metal ions measured in the wine supernatant and juice and wine lees, as well as the limits of quantitation for the different metal ions, are given in Tables S1–S6.

#### 3.4.3. Statistical Analysis

Two-way analyses of variance (ANOVA) with post hoc Tukey tests were performed in GraphPad Prism v8.00 (GraphPad Software Inc., La Jolla, CA, USA) and used to determine significant differences in metal ion concentrations in samples exposed to different oxygen exposure regimes, as well as differences in metal ion concentrations in samples measured at different timepoints. Significant interactions are indicated as follows: not significant (ns) for *p* value > 0.05; * *p* value ≤ 0.05; ** *p* value ≤ 0.01; *** *p* value ≤ 0.001; **** *p* value ≤ 0.0001. PCA plots were used to gain an overview of the data, with all variables mean-centred and scaled prior to evaluation (The Unscrambler X, CAMO Software AS, Oslo, Norway).

## Figures and Tables

**Table 1:** Minimum and maximum concentrations of metal ions measured in this study in Chardonnay wines post-bottling, as well as those of Australian wines and wines from the rest of the world. Limits for the concentrations of the metals for the export of Australian wine are also reported.

Metal Ions	Australian Wines		World Wines		Analytical Requirements for the Export of Australian Wine ^2,6^	Chardonnay Wine Average ^7^
	Minimum ^2,3^	Maximum ^2,3^	Minimum ^2,4^	Maximum ^2,4^		
Al ^1^	ND ^8^	1788	10	9500 ^4^	8000	388
Ca	27	188	3.5	241 ^5^	300	59
Co	0.70	37	n/a ^9^	n/a	20	6
Cr	41	156	ND	1600 ^4^	n/a	16
Cu	1.3	1893	ND	6827 ^4^	1000	36
Fe	1.00	5684	61	10700 ^4^	10000	1558
K	178	1911	265	3065 ^5^	1700	583
Mg	55	254	7.8	718 ^5^	n/a	117
Mn	189	3612	ND	10000^4^	2000	610
Na	4.9	299	ND	310 ^5^	500	46
Ni	ND	98	ND	2300 ^4^	n/a	18
Sn	ND	460	n/a	n/a	1000	6
Zn	13	2721	0.017	3029 ^4^	5000	445

^1^ Metals measured in the Chardonnay wines; ^2^ Minimum, maximum and median values as described in Martin et al. [28]; ^3^ All concentrations reported in µg/L except for Ca, K, Mg, and Na which are reported in mg/L; ^4^ Minimum and maximum values for world wines [4]; ^5^ Minimum and maximum values for world wines, fruit wines, and cocktails [5]; Ibanez et al., 2008 for wines, fruit wines, and cocktails and the review by Tariba et al. 2011 for wines; ^6^ Analytical requirements for the export of wines from Australia for Al, Cu, Sn, and Zn (European Union); Ca and K (Mexico); Co and Na (Canada—Ontario); Fe (South Africa); Mn (China) [29]; ^7^ Average values of the metals measured post-bottling in the Chardonnay wines in the current study, reported in µg/kg for all metals except Ca, K, Mg, and Na, which are reported in mg/Kg; ^8^ ND, not detected; ^9^ n/a, not available.

## Supplementary Materials

The following are available online, Table S1: Average and standard deviation (Stdev) values of the metal ion concentrations (mg/Kg) in juice and wine supernatants, Table S2: Average and standard deviation (Stdev) values of the metal ion concentrations (mg/Kg) in juice and wine lees, Table S3a,b: Summary of the significant effects of oxygen exposure during pressing and handling on the metal ion concentrations in juice and wine supernatants, Table S4a,b: Summary of the significant effects of oxygen exposure during pressing and handling on the metal ion concentrations in juice and wine supernatant, Table S5a,b: Summary of the significant effects of oxygen exposure during pressing and handling on the metal ion concentrations in juice and wine lees, Table S6a,b: Summary of the significant effects of oxygen exposure during pressing and handling on the metal ion concentrations in juice and wine lees.
