# Supplementary material for: Changes in Metal Ion Concentrations in a Chardonnay Wine Related to Oxygen Exposure during Vinification
Source: Molecules. 2019 Apr 17;24(8):1523. doi: 10.3390/molecules24081523 (PMC6515052; doi:10.3390/molecules24081523)
Supplement: Supplementary file 1 [file molecules-24-01523-s001.pdf]

Supporting Information for

Changes in metal ion concentrations in a Chardonnay wine related to oxygen exposure during vinification

Marlize Z. Bekker[1*], Martin P. Day[1] and Paul A. Smith[1,2]

[1] The Australian Wine Research Institute, P.O. Box 197, Glen Osmond, South Australia, 5064.; marlize.bekker@awri.com.au

[2] Current address: Wine Australia, PO Box 2733. Kent Town Business Centre Kent Town, SA 5071; paul.smith@wineaustralia.com

* Correspondence: marlize.bekker@awri.com.au; Tel.: +61-8-8313-6600

Table of Contents

	Page
Table S1. Average and standard deviation (Stdev) values of the metal ion concentrations (mg/Kg) in juice and wine supernatants.	3
Table S2. Average and standard deviation (Stdev) values of the metal ion concentrations (mg/Kg) in juice and wine lees.	8
Table S3a. Summary of the significant effects of oxygen exposure during pressing and handling on the metal ion concentrations in juice and wine supernatants.	9
Table S3b. Summary of the significant effects of oxygen exposure during pressing and handling on the metal ion concentrations in juice and wine supernatants.	11
Table S4a. Summary of the significant effects of oxygen exposure during pressing and handling on the metal ion concentrations in juice and wine supernatant.	13
Table S4b. Summary of the significant effects of oxygen exposure during pressing and handling on the metal ion concentrations in juice and wine supernatant.	14
Table S5a. Summary of the significant effects of oxygen exposure during pressing and handling on the metal ion concentrations in juice and wine lees.	15
Table S5b. Summary of the significant effects of oxygen exposure during pressing and handling on the metal ion concentrations in juice and wine lees.	15
Table S6a. Summary of the significant effects of oxygen exposure during pressing and handling on the metal ion concentrations in juice and wine lees.	16
Table S6b. Summary of the significant effects of oxygen exposure during pressing and handling on the metal ion concentrations in juice and wine lees.	16

Table S1. Average and standard deviation (Stdev) values of the metal ion concentrations (mg/Kg) in juice and wine supernatants.

	Inert – Reductive[4]		Inert - Oxidative		Aerobic – Reductive[4]		Aerobic - Oxidative	
**Aluminium[1] (LOQ 0.10 mg/Kg)[2]**	**Average**	**Stdev[5]**	**Average**	**Stdev**	**Average**	**Stdev**	**Average**	**Stdev**
Press start[3]	0.707	0.047	1.267	0.058	1.020	0.069	0.767	0.023
Press end	0.360	0.017	0.437	0.046	0.430	0.010	0.403	0.031
Post Juice Rack	0.437	0.093	0.507	0.183	0.340	0.090	0.407	0.110
After enzyme	0.100	0.000	0.100	0.000	0.100	0.000	0.100	0.000
Ferment Start	0.100	0.000	0.100	0.000	0.107	0.006	0.103	0.006
Bentonite	0.757	0.090	0.773	0.196	0.683	0.085	0.587	0.071
Ferment End	0.193	0.006	0.220	0.010	0.273	0.075	0.227	0.015
SO_2	0.353	0.025	0.257	0.023	0.360	0.139	0.297	0.133
Post Cold Stab.	0.447	0.081	0.383	0.025	0.470	0.087	0.383	0.025
After Bottling	0.383	0.025	0.383	0.023	0.350	0.017	0.433	0.006
**Calcium (LOQ 2.00 mg/Kg)**								
Press start	120.667	2.517	135.000	1.732	123.333	2.309	106.333	1.528
Press end	106.333	0.577	108.667	0.577	100.667	2.517	114.333	5.774
Post Juice Rack	107.000	1.000	106.667	0.577	105.000	1.732	101.000	2.646
After enzyme	101.000	1.732	100.000	2.000	101.000	2.646	97.000	2.646
Ferment Start	101.667	2.082	101.667	2.082	101.333	2.082	101.000	1.732
Bentonite	111.333	0.577	127.667	11.372	113.333	2.309	111.333	1.155
Ferment End	78.333	2.082	77.333	1.528	77.000	1.732	76.333	1.155
SO_2	79.333	2.082	79.667	4.726	80.000	1.000	84.000	11.358
Post Cold Stab.	59.333	0.577	58.000	1.732	61.667	0.577	63.000	6.083
After Bottling	58.667	2.082	57.333	1.155	61.000	3.606	59.667	1.155
**Cobalt (LOQ 0.001 mg/Kg)**								
Press start	0.005	0.000	0.006	0.000	0.005	0.000	0.004	0.000
Press end	0.003	0.000	0.003	0.000	0.003	0.000	0.003	0.000
Post Juice Rack	0.004	0.000	0.004	0.000	0.004	0.000	0.004	0.000
After enzyme	0.004	0.000	0.004	0.000	0.004	0.000	0.003	0.000
Ferment Start	0.004	0.000	0.004	0.000	0.003	0.001	0.003	0.000
Bentonite	0.002	0.001	0.003	0.001	0.003	0.000	0.003	0.000

Ferment End	0.005	0.001	0.005	0.000	0.004	0.000	0.004	0.000
SO_2	0.005	0.000	0.005	0.000	0.005	0.000	0.005	0.001
Post Cold Stab.	0.006	0.000	0.006	0.000	0.006	0.000	0.006	0.000
After Bottling	0.006	0.000	0.006	0.000	0.006	0.001	0.006	0.000
**Chromium (LOQ 0.001 mg/Kg)**								
Press start	0.005	0.003	0.004	0.001	0.003	0.001	0.002	0.000
Press end	0.001	0.001	0.001	0.001	0.001	0.000	0.001	0.000
Post Juice Rack	0.003	0.001	0.004	0.001	0.002	0.001	0.003	0.001
After enzyme	0.002	0.000	0.002	0.001	0.005	0.004	0.002	0.001
Ferment Start	0.002	0.001	0.007	0.006	0.002	0.000	0.003	0.002
Bentonite	0.004	0.001	0.003	0.000	0.003	0.001	0.002	0.000
Ferment End	0.003	0.001	0.003	0.001	0.002	0.001	0.002	0.000
SO_2	0.024	0.007	0.011	0.010	0.009	0.006	0.003	0.002
Post Cold Stab.	0.023	0.006	0.013	0.013	0.009	0.005	0.004	0.001
After Bottling	0.029	0.008	0.016	0.010	0.008	0.002	0.013	0.006
**Copper (LOQ 0.01 mg/Kg)**								
Press start	0.690	0.010	0.693	0.006	0.667	0.012	0.733	0.006
Press end	0.973	0.006	0.970	0.010	0.830	0.026	0.917	0.057
Post Juice Rack	0.667	0.025	0.673	0.035	0.777	0.015	0.783	0.006
After enzyme	0.613	0.012	0.700	0.010	0.740	0.017	0.730	0.017
Ferment Start	0.490	0.017	0.460	0.026	0.527	0.021	0.563	0.015
Bentonite	0.310	0.010	0.227	0.025	0.233	0.015	0.213	0.023
Ferment End	0.087	0.006	0.033	0.006	0.020	0.010	0.013	0.006
SO_2	0.143	0.006	0.023	0.006	0.030	0.000	0.033	0.015
Post Cold Stab.	0.073	0.006	0.020	0.000	0.010	0.000	0.040	0.010
After Bottling	0.080	0.010	0.023	0.006	0.020	0.010	0.020	0.000
**Iron (LOQ 0.10 mg/Kg)**								
Press start	1.020	0.072	1.600	0.000	1.267	0.058	0.893	0.021
Press end	0.440	0.000	0.517	0.029	0.483	0.015	0.563	0.181
Post Juice Rack	0.540	0.082	0.603	0.197	0.410	0.085	0.467	0.136

After enzyme	0.200	0.000	0.183	0.021	0.187	0.006	0.130	0.017
Ferment Start	0.197	0.015	0.210	0.026	0.163	0.006	0.117	0.015
Bentonite	0.710	0.075	0.717	0.117	0.697	0.055	0.627	0.025
Ferment End	0.920	0.080	0.933	0.076	1.020	0.072	0.957	0.059
SO_2	1.133	0.058	1.023	0.068	1.233	0.058	1.133	0.058
Post Cold Stab.	1.500	0.100	1.500	0.173	1.867	0.058	1.567	0.252
After Bottling	1.467	0.058	1.467	0.058	1.600	0.100	1.700	0.000
**Potassium (LOQ 1.00 mg/Kg)**								
Press start	597.071	25.062	678.090	12.469	595.305	13.670	623.700	12.128
Press end	1570.000	10.000	1653.333	5.774	1403.333	101.160	1696.667	115.902
Post Juice Rack	1146.667	40.415	932.667	118.728	1120.000	87.178	1080.000	17.321
After enzyme	1100.000	10.000	1203.333	32.146	1056.667	23.094	1073.333	46.188
Ferment Start	947.000	144.108	1016.667	41.633	1000.667	35.233	1024.333	55.194
Bentonite	906.000	31.097	1021.000	181.199	972.667	27.465	1036.667	28.868
Ferment End	378.333	6.658	392.000	29.462	383.333	45.884	394.000	11.136
SO_2	732.333	16.166	404.333	50.063	727.667	38.940	480.000	65.000
Post Cold Stab.	637.667	14.844	632.000	20.075	630.333	10.786	656.333	33.650
After Bottling	581.000	15.716	597.000	7.937	586.333	14.468	567.667	23.965
**Magnesium (LOQ 1.00 mg/Kg)**								
Press start	114.047	2.113	120.750	0.867	117.700	2.158	106.600	1.483
Press end	115.193	0.372	117.057	1.147	109.963	2.159	120.400	7.467
Post Juice Rack	116.153	0.300	116.457	0.356	112.683	3.846	105.970	2.156
After enzyme	108.473	0.560	107.203	1.353	107.517	2.403	104.790	2.440
Ferment Start	109.710	0.329	107.947	1.861	107.943	1.972	107.607	1.531
Bentonite	120.550	0.200	126.430	11.434	120.693	1.695	119.523	1.328
Ferment End	133.280	1.153	120.983	10.780	109.903	3.302	111.707	0.708
SO_2	117.543	0.820	115.683	0.922	117.370	0.624	116.100	0.539
Post Cold Stab.	121.903	1.142	118.860	2.721	119.297	1.167	118.817	1.803
After Bottling	118.720	0.586	117.000	1.146	114.857	0.354	116.650	0.908
**Manganese (LOQ 0.01 mg/Kg)**								

Press start	0.840	0.010	1.113	0.006	0.923	0.015	0.687	0.006
Press end	0.547	0.006	0.583	0.006	0.530	0.010	0.623	0.042
Post Juice Rack	0.620	0.000	0.643	0.006	0.607	0.015	0.600	0.010
After enzyme	0.577	0.006	0.600	0.000	0.577	0.012	0.567	0.015
Ferment Start	0.573	0.006	0.593	0.006	0.577	0.006	0.580	0.010
Bentonite	0.610	0.000	0.670	0.052	0.603	0.006	0.610	0.000
Ferment End	0.627	0.006	0.667	0.015	0.613	0.021	0.637	0.012
SO_2	0.663	0.006	0.677	0.012	0.663	0.006	0.660	0.017
Post Cold Stab.	0.627	0.006	0.643	0.012	0.620	0.010	0.620	0.010
After Bottling	0.617	0.012	0.627	0.006	0.597	0.006	0.600	0.000
**Sodium (LOQ 1.00 mg/Kg)**								
Press start	27.977	0.990	32.537	3.071	30.723	1.972	56.610	1.596
Press end	26.293	0.099	28.300	4.632	24.650	0.383	40.583	2.559
Post Juice Rack	19.923	0.558	19.967	0.155	19.720	0.798	18.200	0.333
After enzyme	18.583	0.536	18.113	0.122	18.743	0.528	18.017	0.396
Ferment Start	20.490	0.173	19.287	0.408	22.007	0.461	21.390	0.748
Bentonite	24.647	0.304	28.250	3.700	26.013	0.137	25.787	0.508
Ferment End	26.723	0.636	27.207	0.680	26.653	1.272	27.550	0.581
SO_2	28.493	0.597	31.390	2.160	29.267	0.720	28.660	0.220
Post Cold Stab.	47.327	0.566	45.230	1.717	46.820	0.716	44.610	2.200
After Bottling	46.937	1.119	45.357	0.598	44.417	2.389	46.750	0.243
**Nickel (LOQ 0.01 mg/Kg)**								
Press start	0.010	0.000	0.010	0.000	0.010	0.000	0.010	0.000
Press end	0.050	0.069	0.010	0.000	0.010	0.000	0.010	0.000
Post Juice Rack	0.010	0.000	0.010	0.000	0.010	0.000	0.010	0.000
After enzyme	0.010	0.000	0.010	0.000	0.010	0.000	0.010	0.000
Ferment Start	0.010	0.000	0.010	0.000	0.010	0.000	0.010	0.000
Bentonite	0.010	0.000	0.013	0.006	0.017	0.012	0.010	0.000
Ferment End	0.013	0.006	0.013	0.006	0.010	0.000	0.010	0.000
SO_2	0.010	0.000	0.010	0.000	0.010	0.000	0.010	0.000
Post Cold Stab.	0.010	0.000	0.013	0.006	0.010	0.000	0.010	0.000

After Bottling	0.020	0.000	0.017	0.006	0.013	0.006	0.020	0.000
**Tin (LOQ 0.002 mg/Kg)**								
Press start	0.003	0.001	0.004	0.002	0.002	0.000	0.006	0.006
Press end	0.005	0.002	0.003	0.001	0.006	0.003	0.003	0.000
Post Juice Rack	0.003	0.001	0.009	0.002	0.003	0.001	0.002	0.001
After enzyme	0.005	0.005	0.002	0.000	0.002	0.000	0.003	0.001
Ferment Start	0.002	0.000	0.003	0.001	0.003	0.001	0.003	0.002
Bentonite	0.002	0.001	0.002	0.001	0.002	0.000	0.002	0.000
Ferment End	0.003	0.001	0.002	0.000	0.004	0.003	0.002	0.000
SO_2	0.002	0.000	0.002	0.000	0.002	0.000	0.002	0.000
Post Cold Stab.	0.002	0.000	0.002	0.000	0.002	0.000	0.002	0.000
After Bottling	0.006	0.002	0.006	0.002	0.003	0.001	0.008	0.003
**Zinc (LOQ 0.01 mg/Kg)**								
Press start	0.480	0.017	0.577	0.006	0.520	0.000	0.370	0.010
Press end	0.397	0.006	0.370	0.010	0.343	0.032	0.393	0.025
Post Juice Rack	0.403	0.006	0.417	0.047	0.427	0.029	0.380	0.000
After enzyme	0.393	0.015	0.367	0.006	0.407	0.023	0.387	0.006
Ferment Start	0.300	0.036	0.267	0.023	0.273	0.035	0.190	0.026
Bentonite	0.040	0.010	0.107	0.083	0.060	0.010	0.067	0.035
Ferment End	0.367	0.006	0.387	0.038	0.345	0.005	0.280	0.026
SO_2	0.437	0.021	0.450	0.053	0.440	0.000	0.440	0.079
Post Cold Stab.	0.410	0.000	0.390	0.017	0.420	0.010	0.393	0.006
After Bottling	0.467	0.032	0.430	0.010	0.437	0.031	0.447	0.015

[1]Element analysed using ICP-MS; [2] LOQ: limit of quantitation; [3]Analysis point during vinification; [4]Oxygen exposure regime; [5]STDEV: standard deviation.

Table S2. Average and standard deviation (Stdev) values of the metal ion concentrations (mg/Kg) in juice and wine lees.

	Inert – Reductive[4]		Inert - Oxidative		Aerobic - Reductive		Aerobic - Oxidative	
**Aluminium[1] (LOQ 5.00 mg/Kg)[2]**	**Average**	**Stdev[5]**	**Average**	**Stdev**	**Average**	**Stdev**	**Average**	**Stdev**
Juice Lees[3]	237.84	63.86	198.67	53.52	239.00	36.50	228.23	35.99
Wine Lees	462.79	103.60	348.58	48.73	458.75	86.92	351.44	39.28
**Calcium (LOQ 100 mg/Kg)**								
Juice Lees	523.95	94.72	403.50	84.90	483.91	10.39	453.95	84.57
Wine Lees	2551.34	1215.37	4157.32	839.87	2793.52	1406.12	2685.58	1488.70
**Cobalt (LOQ 0.05 mg/Kg)**								
Juice Lees	0.08	0.02	0.07	0.02	0.09	0.01	0.09	0.02
Wine Lees	0.29	0.08	0.24	0.04	0.28	0.06	0.26	0.05
**Chromium (LOQ 0.05 mg/Kg)**								
Juice Lees	0.56	0.15	0.45	0.12	0.53	0.09	0.55	0.13
Wine Lees	0.41	0.02	0.41	0.02	0.40	0.04	0.41	0.04
**Copper (LOQ 0.50 mg/Kg)**								
Juice Lees	59.07	14.68	36.97	8.39	30.81	0.15	25.99	1.13
Wine Lees	94.83	8.07	123.89	18.26	207.06	5.02	198.33	18.40
**Iron (LOQ 5.00 mg/Kg)**								
Juice Lees	309.58	67.12	268.51	67.05	320.20	34.84	317.66	48.08
Wine Lees	357.61	47.50	304.94	38.75	325.34	46.96	294.78	65.60
**Potassium (LOQ 50.0 mg/Kg)**								
Juice Lees	21719.45	11485.87	16621.74	11625.48	19640.77	6482.17	24295.89	2517.38
Wine Lees	19860.91	5789.87	32423.14	13189.56	20496.99	10933.66	17959.79	11128.36
**Magnesium (LOQ 50.0 mg/Kg)**								
Juice Lees	290.67	17.61	242.48	14.18	317.40	36.60	181.19	14.65
Wine Lees	496.02	215.12	354.83	114.96	466.92	168.79	494.85	80.59
**Manganese (LOQ 5.00 mg/Kg)**								
Juice Lees	17.78	3.05	13.34	3.21	15.99	1.91	15.57	1.93
Wine Lees	10.75	1.22	10.41	1.66	10.33	1.58	11.84	1.06
**Sodium (LOQ 50.0 mg/Kg)**								

Juice Lees	50.31	18.87	29.44	0.58	39.59	7.73	16.93	2.29
Wine Lees	65.46	59.29	31.90	14.56	68.61	46.50	46.17	18.87
**Nickel (LOQ 0.50 mg/Kg)**								
Juice Lees	0.50	0.00	0.50	0.00	0.50	0.00	0.50	0.00
Wine Lees	0.74	0.14	0.57	0.07	0.69	0.11	0.62	0.08
**Zinc (LOQ 0.50 mg/Kg)**								
Juice Lees	3.84	0.86	2.45	0.35	3.55	0.31	3.01	0.90
Wine Lees	10.59	3.55	9.33	2.67	14.26	4.42	14.85	3.50

[1]Element analysed using ICP-MS; [2] LOQ: limit of quantitation; [3]Analysis point during vinification; [4]Oxygen exposure regime; [5]STDEV: standard deviation.

Table S3a. Summary of the significant effects of oxygen exposure during pressing and handling on the metal ion concentrations in juice and wine supernatants.

	**Aluminium**		**Calcium**		**Cobalt**		**Chromium**		**Copper**		**Iron**	
**Tukey's multiple comparisons test**	**Adjusted P Value**		**Adjusted P Value**		**Adjusted P Value**		**Adjusted P Value**		**Adjusted P Value**		**Adjusted P Value**	
**Press start**												
IR vs. IO[1]	****	<0.0001	****	<0.0001	****	<0.0001	ns	0.9997	ns	0.9950	****	<0.0001
IR vs. AR	****	<0.0001	ns	0.7766	ns	>0.9999	ns	0.9798	ns	0.3329	**	0.0042
IR vs. AO	ns	0.7221	****	<0.0001	****	<0.0001	ns	0.8639	*	0.0121	ns	0.2825
IO vs. AR	***	0.0003	***	0.0004	****	<0.0001	ns	0.9913	ns	0.2208	****	<0.0001
IO vs. AO	****	<0.0001	****	<0.0001	****	<0.0001	ns	0.9039	*	0.0240	****	<0.0001
AR vs. AO	***	0.0002	****	<0.0001	****	<0.0001	ns	0.9798	****	<0.0001	****	<0.0001
**Press end**												
IR vs. IO	ns	0.5415	ns	0.8384	ns	>0.9999	ns	>0.9999	ns	0.9950	ns	0.6982
IR vs. AR	ns	0.6148	ns	0.1879	ns	>0.9999	ns	0.9997	****	<0.0001	ns	0.9271
IR vs. AO	ns	0.8736	*	0.0274	ns	>0.9999	ns	0.9997	***	0.0005	ns	0.3054
IO vs. AR	ns	0.9994	*	0.0274	ns	>0.9999	ns	0.9997	****	<0.0001	ns	0.9648
IO vs. AO	ns	0.9372	ns	0.1879	ns	>0.9999	ns	0.9997	**	0.0012	ns	0.9110
AR vs. AO	ns	0.9664	****	<0.0001	ns	>0.9999	ns	>0.9999	****	<0.0001	ns	0.6693
**Post Juice Rack**												
IR vs. IO	ns	0.6148	ns	0.9994	ns	>0.9999	ns	0.9997	ns	0.9624	ns	0.8057
IR vs. AR	ns	0.3369	ns	0.8911	ns	>0.9999	ns	0.9913	****	<0.0001	ns	0.2607
IR vs. AO	ns	0.9531	ns	0.1485	ns	>0.9999	ns	0.9974	****	<0.0001	ns	0.7264
IO vs. AR	*	0.0237	ns	0.9332	ns	>0.9999	ns	0.9798	****	<0.0001	*	0.0370
IO vs. AO	ns	0.3072	ns	0.1879	ns	>0.9999	ns	0.9913	****	<0.0001	ns	0.2204
AR vs. AO	ns	0.6512	ns	0.4854	ns	>0.9999	ns	0.9997	ns	0.9624	ns	0.8525

**After enzyme**												
IR vs. IO	ns	>0.9999	ns	0.9843	ns	>0.9999	ns	0.9997	****	<0.0001	ns	0.9953
IR vs. AR	ns	>0.9999	ns	>0.9999	ns	>0.9999	ns	0.8639	****	<0.0001	ns	0.9976
IR vs. AO	ns	>0.9999	ns	0.4854	****	<0.0001	ns	0.9997	****	<0.0001	ns	0.7539
IO vs. AR	ns	>0.9999	ns	0.9843	ns	>0.9999	ns	0.8172	*	0.0240	ns	>0.9999
IO vs. AO	ns	>0.9999	ns	0.7078	****	<0.0001	ns	0.9974	ns	0.1378	ns	0.8736
AR vs. AO	ns	>0.9999	ns	0.4854	****	<0.0001	ns	0.9039	ns	0.8862	ns	0.8525
**Ferment Start**												
IR vs. IO	ns	>0.9999	ns	>0.9999	ns	>0.9999	ns	0.5875	ns	0.1378	ns	0.9976
IR vs. AR	ns	0.9994	ns	0.9994	**	0.0026	ns	0.9997	*	0.0452	ns	0.9648
IR vs. AO	ns	>0.9999	ns	0.9952	****	<0.0001	ns	0.9974	****	<0.0001	ns	0.6693
IO vs. AR	ns	0.9994	ns	0.9994	**	0.0026	ns	0.5263	****	<0.0001	ns	0.9110
IO vs. AO	ns	>0.9999	ns	0.9952	****	<0.0001	ns	0.7083	****	<0.0001	ns	0.5506
AR vs. AO	ns	>0.9999	ns	0.9994	ns	0.2690	ns	0.9913	*	0.0452	ns	0.9110
**Bentonite**												
IR vs. IO	ns	0.9914	****	<0.0001	ns	0.2690	ns	0.9974	****	<0.0001	ns	0.9997
IR vs. AR	ns	0.5781	ns	0.8911	**	0.0026	ns	0.9913	****	<0.0001	ns	0.9976
IR vs. AO	*	0.0202	ns	>0.9999	**	0.0026	ns	0.9619	****	<0.0001	ns	0.6399
IO vs. AR	ns	0.4008	****	<0.0001	ns	0.2690	ns	0.9997	ns	0.9624	ns	0.9920
IO vs. AO	**	0.0088	****	<0.0001	ns	0.2690	ns	0.9913	ns	0.7676	ns	0.5804
AR vs. AO	ns	0.3369	ns	0.8911	ns	>0.9999	ns	0.9974	ns	0.4705	ns	0.7539
**Ferment End**												
IR vs. IO	ns	0.9664	ns	0.9843	ns	0.2690	ns	>0.9999	**	0.0012	ns	0.9976
IR vs. AR	ns	0.5052	ns	0.9641	**	0.0026	ns	0.9997	****	<0.0001	ns	0.4917
IR vs. AO	ns	0.9372	ns	0.8911	**	0.0026	ns	0.9974	****	<0.0001	ns	0.9540
IO vs. AR	ns	0.7883	ns	0.9994	****	<0.0001	ns	0.9997	ns	0.7676	ns	0.6102
IO vs. AO	ns	0.9994	ns	0.9843	****	<0.0001	ns	0.9974	ns	0.4705	ns	0.9874
AR vs. AO	ns	0.8474	ns	0.9952	ns	>0.9999	ns	0.9997	ns	0.9624	ns	0.8057
**SO_2**												
IR vs. IO	ns	0.3369	ns	0.9994	ns	>0.9999	***	0.0008	****	<0.0001	ns	0.4071
IR vs. AR	ns	0.9994	ns	0.9952	ns	>0.9999	***	0.0002	****	<0.0001	ns	0.4917
IR vs. AO	ns	0.7559	ns	0.3479	ns	0.2690	****	<0.0001	****	<0.0001	ns	>0.9999
IO vs. AR	ns	0.2791	ns	0.9994	ns	>0.9999	ns	0.9798	ns	0.9624	*	0.0196
IO vs. AO	ns	0.8974	ns	0.4143	ns	0.2690	ns	0.1490	ns	0.8862	ns	0.4071
AR vs. AO	ns	0.6871	ns	0.4854	ns	0.2690	ns	0.3041	ns	0.9950	ns	0.4917
**Post Cold Stab.**												
IR vs. IO	ns	0.6871	ns	0.9641	ns	>0.9999	*	0.0134	**	0.0012	ns	>0.9999
IR vs. AR	ns	0.9770	ns	0.8384	ns	>0.9999	***	0.0004	****	<0.0001	****	<0.0001
IR vs. AO	ns	0.6871	ns	0.5596	ns	>0.9999	****	<0.0001	ns	0.0811	ns	0.7804
IO vs. AR	ns	0.4346	ns	0.5596	ns	>0.9999	ns	0.7083	ns	0.8862	****	<0.0001
IO vs. AO	ns	>0.9999	ns	0.2877	ns	>0.9999	*	0.0495	ns	0.4705	ns	0.7804

AR vs. AO	ns	0.4346	ns	0.9641	ns	>0.9999	ns	0.4087	ns	0.1378	***	0.0003
**After Bottling**												
IR vs. IO	ns	>0.9999	ns	0.9641	ns	>0.9999	**	0.0016	***	0.0005	ns	>0.9999
IR vs. AR	ns	0.9372	ns	0.8384	ns	0.2690	****	<0.0001	***	0.0002	ns	0.2400
IR vs. AO	ns	0.8188	ns	0.9843	ns	>0.9999	***	0.0001	***	0.0002	**	0.0075
IO vs. AR	ns	0.9372	ns	0.5596	ns	0.2690	ns	0.1217	ns	0.9950	ns	0.2400
IO vs. AO	ns	0.8188	ns	0.8384	ns	>0.9999	ns	0.9039	ns	0.9950	**	0.0075
AR vs. AO	ns	0.4695	ns	0.9641	ns	0.2690	ns	0.4087	ns	>0.9999	ns	0.4917

[1] The four combinations of oxygen exposure regimes during pressing and handling: inert-reductive (IR), inert-oxidative (IO), aerobic-reductive (AR), and aerobic-oxidative (AO).

Table S3b. Summary of the significant effects of oxygen exposure during pressing and handling on the metal ion concentrations in juice and wine supernatants.

	Potassium		Magnesium		Manganese		Sodium		Tin		Zinc	
**Tukey's multiple comparisons test**	**Adjusted P Value**		**Adjusted P Value**		**Adjusted P Value**		**Adjusted P Value**		**Adjusted P Value**		**Adjusted P Value**	
**Press start**												
IR vs. IO[1]	ns	0.3069	ns	0.0534	****	<0.0001	**	0.0016	ns	0.7443	***	0.0005
IR vs. AR	ns	>0.9999	ns	0.4936	****	<0.0001	ns	0.1088	ns	0.9577	ns	0.3243
IR vs. AO	ns	0.9396	*	0.0254	****	<0.0001	****	<0.0001	ns	0.1135	****	<0.0001
IO vs. AR	ns	0.2884	ns	0.6402	****	<0.0001	ns	0.4351	ns	0.4339	ns	0.0805
IO vs. AO	ns	0.6459	****	<0.0001	****	<0.0001	****	<0.0001	ns	0.5897	****	<0.0001
AR vs. AO	ns	0.9280	***	0.0003	****	<0.0001	****	<0.0001	*	0.0339	****	<0.0001
**Press end**												
IR vs. IO	ns	0.2827	ns	0.8881	*	0.0106	ns	0.3444	ns	0.4339	ns	0.6653
IR vs. AR	**	0.0031	ns	0.1871	ns	0.4709	ns	0.5214	ns	0.9577	ns	0.1108
IR vs. AO	*	0.0382	ns	0.1903	****	<0.0001	****	<0.0001	ns	0.4339	ns	0.9990
IO vs. AR	****	<0.0001	*	0.0365	****	<0.0001	*	0.0164	ns	0.1897	ns	0.6653
IO vs. AO	ns	0.7867	ns	0.5686	**	0.0044	****	<0.0001	ns	>0.9999	ns	0.7506
AR vs. AO	****	<0.0001	***	0.0007	****	<0.0001	****	<0.0001	ns	0.1897	ns	0.1495
**Post Juice Rack**												
IR vs. IO	****	<0.0001	ns	0.9994	ns	0.1845	ns	>0.9999	****	<0.0001	ns	0.9405
IR vs. AR	ns	0.9393	ns	0.5378	ns	0.6524	ns	0.9983	ns	>0.9999	ns	0.7506
IR vs. AO	ns	0.4803	***	0.0010	ns	0.3086	ns	0.4802	ns	0.9943	ns	0.7506
IO vs. AR	***	0.0007	ns	0.4652	*	0.0106	ns	0.9969	****	<0.0001	ns	0.9735
IO vs. AO	*	0.0112	***	0.0006	**	0.0017	ns	0.4583	****	<0.0001	ns	0.4019
AR vs. AO	ns	0.8242	ns	0.0529	ns	0.9375	ns	0.5861	ns	0.9943	ns	0.1977
**After enzyme**												
IR vs. IO	ns	0.1247	ns	0.9607	ns	0.1845	ns	0.9794	ns	0.1897	ns	0.6653
IR vs. AR	ns	0.7867	ns	0.9825	ns	>0.9999	ns	0.9991	ns	0.1897	ns	0.9405

IR vs. AO	ns	0.9393	ns	0.4865	ns	0.8195	ns	0.9649	ns	0.4339	ns	0.9918
IO vs. AR	*	0.0117	ns	0.9994	ns	0.1845	ns	0.9527	ns	>0.9999	ns	0.3243
IO vs. AO	*	0.0317	ns	0.7862	*	0.0241	ns	0.9998	ns	0.9577	ns	0.8274
AR vs. AO	ns	0.9840	ns	0.7168	ns	0.8195	ns	0.9299	ns	0.9577	ns	0.8274
**Ferment Start**												
IR vs. IO	ns	0.4413	ns	0.9032	ns	0.3086	ns	0.7478	ns	0.8733	ns	0.4867
IR vs. AR	ns	0.6556	ns	0.9027	ns	0.9914	ns	0.5878	ns	0.8733	ns	0.6653
IR vs. AO	ns	0.3479	ns	0.8473	ns	0.9375	ns	0.8762	ns	0.7443	****	<0.0001
IO vs. AR	ns	0.9858	ns	>0.9999	ns	0.4709	ns	0.1142	ns	>0.9999	ns	0.9918
IO vs. AO	ns	0.9984	ns	0.9992	ns	0.6524	ns	0.3031	ns	0.9943	**	0.0082
AR vs. AO	ns	0.9565	ns	0.9992	ns	0.9914	ns	0.9555	ns	0.9943	**	0.0034
**Bentonite**												
IR vs. IO	ns	0.0710	ns	0.1118	****	<0.0001	*	0.0182	ns	>0.9999	*	0.0277
IR vs. AR	ns	0.4803	ns	>0.9999	ns	0.9375	ns	0.6662	ns	0.9943	ns	0.8274
IR vs. AO	*	0.0305	ns	0.9785	ns	>0.9999	ns	0.7776	ns	0.9943	ns	0.6653
IO vs. AR	ns	0.7255	ns	0.1260	****	<0.0001	ns	0.2512	ns	0.9943	ns	0.1977
IO vs. AO	ns	0.9866	*	0.0439	****	<0.0001	ns	0.1772	ns	0.9943	ns	0.3243
AR vs. AO	ns	0.5158	ns	0.9688	ns	0.9375	ns	0.9976	ns	>0.9999	ns	0.9918
**Ferment End**												
IR vs. IO	ns	0.9910	****	<0.0001	**	0.0044	ns	0.9777	ns	0.9577	ns	0.8274
IR vs. AR	ns	0.9995	****	<0.0001	ns	0.6524	ns	>0.9999	ns	0.8733	ns	0.7903
IR vs. AO	ns	0.9866	****	<0.0001	ns	0.8195	ns	0.9009	ns	0.9577	**	0.0021
IO vs. AR	ns	0.9977	***	0.0003	****	<0.0001	ns	0.9672	ns	0.5897	ns	0.2889
IO vs. AO	ns	>0.9999	**	0.0031	ns	0.0512	ns	0.9918	ns	>0.9999	***	0.0001
AR vs. AO	ns	0.9957	ns	0.8973	ns	0.1845	ns	0.8774	ns	0.5897	*	0.0334
**SO_2**												
IR vs. IO	****	<0.0001	ns	0.8886	ns	0.6524	ns	0.0823	ns	>0.9999	ns	0.9405
IR vs. AR	ns	0.9996	ns	0.9999	ns	>0.9999	ns	0.9170	ns	>0.9999	ns	0.9990
IR vs. AO	****	<0.0001	ns	0.9438	ns	0.9914	ns	0.9990	ns	>0.9999	ns	0.9990
IO vs. AR	****	<0.0001	ns	0.9140	ns	0.6524	ns	0.2949	ns	>0.9999	ns	0.9735
IO vs. AO	ns	0.3673	ns	0.9985	ns	0.4709	ns	0.1122	ns	>0.9999	ns	0.9735
AR vs. AO	****	<0.0001	ns	0.9607	ns	0.9914	ns	0.9575	ns	>0.9999	ns	>0.9999
**Post Cold Stab.**												
IR vs. IO	ns	0.9993	ns	0.6418	ns	0.4709	ns	0.3059	ns	>0.9999	ns	0.8274
IR vs. AR	ns	0.9986	ns	0.7441	ns	0.9375	ns	0.9745	ns	>0.9999	ns	0.9735
IR vs. AO	ns	0.9778	ns	0.6313	ns	0.9375	ns	0.1149	ns	>0.9999	ns	0.8916
IO vs. AR	ns	>0.9999	ns	0.9983	ns	0.1845	ns	0.5493	ns	>0.9999	ns	0.5758
IO vs. AO	ns	0.9529	ns	>0.9999	ns	0.1845	ns	0.9548	ns	>0.9999	ns	0.9990
AR vs. AO	ns	0.9434	ns	0.9977	ns	>0.9999	ns	0.2611	ns	>0.9999	ns	0.6653
**After Bottling**												
IR vs. IO	ns	0.9858	ns	0.9094	ns	0.8195	ns	0.5545	ns	0.9577	ns	0.4019

IR vs. AR	ns	0.9995	ns	0.4443	ns	0.3086	ns	0.1614	ns	0.2969	ns	0.5758
IR vs. AO	ns	0.9917	ns	0.8533	ns	0.4709	ns	0.9986	ns	0.4339	ns	0.8274
IO vs. AR	ns	0.9957	ns	0.8399	ns	0.0512	ns	0.8615	ns	0.1135	ns	0.9918
IO vs. AO	ns	0.9213	ns	0.9991	ns	0.1011	ns	0.6524	ns	0.7443	ns	0.8916
AR vs. AO	ns	0.9778	ns	0.8988	ns	0.9914	ns	0.2175	**	0.0082	ns	0.9735

[1] The four combinations of oxygen exposure regimes during pressing and handling: inert-reductive (IR), inert-oxidative (IO), aerobic-reductive (AR), and aerobic-oxidative (AO).

Table S4a. Summary of the significant effects of oxygen exposure during pressing and handling on the metal ion concentrations in juice and wine supernatant.

	Aluminium		Calcium		Cobalt		Chromium		Copper		Iron	
**Tukey's multiple comparisons test**	**Adjusted P Value**		**Adjusted P Value**		**Adjusted P Value**		**Adjusted P Value**		**Adjusted P Value**		**Adjusted P Value**	
**Press conditions: Inert - Aerobic**												
Press start	ns	0.7149	***	0.0002	****	<0.0001	ns	0.9992	ns	>0.9999	*	0.0299
Press end	ns	>0.9999	ns	>0.9999	ns	>0.9999	ns	>0.9999	ns	0.9992	ns	0.9997
Post Juice Rack	ns	0.6503	ns	0.8692	ns	>0.9999	ns	>0.9999	ns	0.9979	ns	0.5711
After enzyme	ns	>0.9999	ns	0.9999	ns	0.0541	ns	0.9996	ns	0.9999	ns	>0.9999
Ferment Start	ns	>0.9999	ns	>0.9999	****	<0.0001	ns	0.9983	ns	>0.9999	ns	0.9944
Bentonite	ns	0.2653	ns	0.1316	ns	0.0541	ns	>0.9999	ns	>0.9999	ns	0.9990
Ferment End	ns	0.9981	ns	>0.9999	****	<0.0001	ns	>0.9999	ns	>0.9999	ns	0.9955
SO2 and rack	ns	>0.9999	ns	0.9923	ns	0.9859	**	0.0011	ns	>0.9999	ns	0.8418
Post Cold Stab	ns	>0.9999	ns	0.8975	ns	>0.9999	***	0.0005	*	0.0340	*	0.0500
After Bottling	ns	>0.9999	ns	0.9955	ns	0.9859	***	0.0007	ns	>0.9999	ns	0.7409
**Handling conditions: Reductive - Oxidative**												
Press start	ns	0.0988	ns	>0.9999	ns	>0.9999	ns	>0.9999	ns	>0.9999	ns	0.8999
Press end	ns	>0.9999	ns	0.1035	ns	>0.9999	ns	>0.9999	ns	>0.9999	ns	0.9835
Post Juice Rack	ns	0.9414	ns	0.9987	ns	>0.9999	ns	>0.9999	ns	>0.9999	ns	0.9979
After enzyme	ns	>0.9999	ns	0.9956	ns	0.3226	ns	>0.9999	ns	>0.9999	ns	>0.9999
Ferment Start	ns	>0.9999	ns	>0.9999	ns	0.9987	ns	0.9937	ns	>0.9999	ns	>0.9999
Bentonite	ns	0.9990	ns	0.2001	ns	0.9987	ns	>0.9999	ns	>0.9999	ns	>0.9999
Ferment End	ns	>0.9999	ns	>0.9999	ns	0.9987	ns	>0.9999	ns	>0.9999	ns	>0.9999
SO_2	ns	0.8554	ns	0.9987	ns	0.9987	*	0.0224	ns	>0.9999	ns	0.8905
Post Cold Stab	ns	0.8980	ns	>0.9999	ns	>0.9999	ns	0.1164	*	0.0289	ns	0.5021
After Bottling	ns	0.9985	ns	>0.9999	ns	0.9987	ns	0.9215	ns	>0.9999	ns	0.8110

Table S4b. Summary of the significant effects of oxygen exposure during pressing and handling on the metal ion concentrations in juice and wine supernatant.

	Potassium		Magnesium		Manganese		Sodium		Tin		Zinc	
Tukey's multiple comparisons test	Adjusted P Value		Adjusted P Value		Adjusted P Value		Adjusted P Value		Adjusted P Value		Adjusted P Value	
**Press conditions: Inert - Aerobic**												
Press start	ns	0.9998	ns	0.2567	****	<0.0001	****	<0.0001	ns	>0.9999	ns	0.0740
Press end	ns	0.9237	ns	>0.9999	ns	>0.9999	ns	0.3185	ns	>0.9999	ns	>0.9999
Post Juice Rack	ns	0.9332	*	0.0176	ns	0.9744	ns	0.9999	*	0.0280	ns	>0.9999
After enzyme	ns	0.6131	ns	0.9994	ns	0.9996	ns	>0.9999	ns	0.9886	ns	0.9999
Ferment Start	ns	0.9996	ns	>0.9999	ns	>0.9999	ns	0.9915	ns	0.9996	ns	0.6300
Bentonite	ns	0.9956	ns	0.8179	ns	0.9265	ns	0.9977	ns	>0.9999	ns	>0.9999
Ferment End	ns	>0.9999	****	<0.0001	ns	0.9966	ns	>0.9999	ns	>0.9999	ns	>0.9999
SO_2	ns	0.8287	ns	>0.9999	ns	>0.9999	ns	>0.9999	ns	>0.9999	ns	>0.9999
Post Cold Stab	ns	>0.9999	ns	0.9998	ns	0.9999	ns	>0.9999	ns	>0.9999	ns	>0.9999
After Bottling	ns	>0.9999	ns	0.9933	ns	0.9939	ns	>0.9999	ns	>0.9999	ns	>0.9999
**Handling conditions: Reductive - Oxidative**												
Press start	ns	0.8218	ns	0.9976	ns	>0.9999	****	<0.0001	ns	0.1418	ns	0.9912
Press end	****	<0.0001	ns	0.2953	ns	0.3526	***	0.0006	ns	0.2090	ns	>0.9999
Post Juice Rack	*	0.0137	ns	0.9590	ns	>0.9999	ns	>0.9999	*	0.0372	ns	0.9998
After enzyme	ns	0.7313	ns	0.9994	ns	>0.9999	ns	>0.9999	ns	0.9805	ns	0.9969
Ferment Start	ns	0.9261	ns	>0.9999	ns	>0.9999	ns	>0.9999	ns	0.9992	ns	0.4322
Bentonite	ns	0.2028	ns	0.9956	ns	0.9695	ns	0.8929	ns	>0.9999	ns	0.9221
Ferment End	ns	>0.9999	ns	0.5432	ns	0.9786	ns	>0.9999	ns	0.9449	ns	0.0748
SO_2	****	<0.0001	ns	0.9994	ns	>0.9999	ns	0.8092	ns	>0.9999	ns	>0.9999
Post Cold Stab	ns	>0.9999	ns	0.9997	ns	>0.9999	ns	0.9893	ns	>0.9999	ns	0.9969
After Bottling	ns	>0.9999	ns	>0.9999	ns	>0.9999	ns	>0.9999	ns	0.1418	ns	>0.9999

Table S5a. Summary of the significant effects of oxygen exposure during pressing and handling on the metal ion concentrations in juice and wine lees.

	Aluminium	Calcium	Cobalt	Chromium	Copper	Iron
Tukey's multiple comparisons test	Adjusted P Value	Adjusted P Value	Adjusted P Value	Adjusted P Value	Adjusted P Value	Adjusted P Value
**Juice Lees**						

IR vs. IO[1]	ns	0.8706	ns	0.9983	ns	0.9897	ns	0.4953	ns	0.1252	ns	0.7828
IR vs. AR	ns	>0.9999	ns	>0.9999	ns	0.9998	ns	0.9831	*	0.0366	ns	0.9947
IR vs. AO	ns	0.9976	ns	0.9997	ns	0.9995	ns	0.9996	*	0.0132	ns	0.9976
IO vs. AR	ns	0.8607	ns	0.9995	ns	0.9800	ns	0.7091	ns	0.9108	ns	0.6439
IO vs. AO	ns	0.9383	ns	0.9999	ns	0.9753	ns	0.5553	ns	0.6513	ns	0.6784
AR vs. AO	ns	0.9966	ns	>0.9999	ns	>0.9999	ns	0.9936	ns	0.9544	ns	>0.9999
**Wine Lees**												
IR vs. IO	ns	0.1601	ns	0.1657	ns	0.4128	ns	>0.9999	*	0.0310	ns	0.6305
IR vs. AR	ns	0.9998	ns	0.9869	ns	0.9945	ns	0.9972	****	<0.0001	ns	0.8793
IR vs. AO	ns	0.1757	ns	0.9977	ns	0.8013	ns	>0.9999	****	<0.0001	ns	0.4932
IO vs. AR	ns	0.1824	ns	0.2801	ns	0.5518	ns	0.9990	****	<0.0001	ns	0.9650
IO vs. AO	ns	>0.9999	ns	0.2234	ns	0.9030	ns	>0.9999	****	<0.0001	ns	0.9954
AR vs. AO	ns	0.1997	ns	0.9988	ns	0.9103	ns	0.9982	ns	0.7876	ns	0.8951

[1] The four combinations of oxygen exposure regimes during pressing and handling: inert-reductive (IR), inert-oxidative (IO), aerobic-reductive (AR), and aerobic-oxidative (AO).

Table S5b. Summary of the significant effects of oxygen exposure during pressing and handling on the metal ion concentrations in juice and wine lees.

	**Potassium**		**Magnesium**		**Manganese**		**Sodium**		**Nickel**		**Zinc**	
**Tukey's multiple comparisons test**	**Adjusted P Value**		**Adjusted P Value**		**Adjusted P Value**		**Adjusted P Value**		**Adjusted P Value**		**Adjusted P Value**	
**Juice Lees**												
IR vs. IO[1]	ns	0.9182	ns	0.9486	ns	0.0807	ns	0.8124	ns	>0.9999	ns	0.9091
IR vs. AR	ns	0.9936	ns	0.9904	ns	0.7227	ns	0.9677	ns	>0.9999	ns	0.9990
IR vs. AO	ns	0.9880	ns	0.6233	ns	0.5789	ns	0.5078	ns	>0.9999	ns	0.9784
IO vs. AR	ns	0.9810	ns	0.8370	ns	0.4299	ns	0.9724	ns	>0.9999	ns	0.9522
IO vs. AO	ns	0.7733	ns	0.9019	ns	0.5690	ns	0.9503	ns	>0.9999	ns	0.9929
AR vs. AO	ns	0.9359	ns	0.4500	ns	0.9947	ns	0.7727	ns	>0.9999	ns	0.9940
**Wine Lees**												
IR vs. IO	ns	0.4210	ns	0.4199	ns	0.9971	ns	0.5034	*	0.0479	ns	0.9313
IR vs. AR	ns	0.9998	ns	0.9878	ns	0.9947	ns	0.9991	ns	0.7867	ns	0.3362
IR vs. AO	ns	0.9951	ns	>0.9999	ns	0.9165	ns	0.8449	ns	0.1920	ns	0.2214
IO vs. AR	ns	0.4645	ns	0.6060	ns	>0.9999	ns	0.4289	ns	0.2479	ns	0.1306
IO vs. AO	ns	0.3048	ns	0.4269	ns	0.8343	ns	0.9289	ns	0.8651	ns	0.0789
AR vs. AO	ns	0.9885	ns	0.9891	ns	0.8114	ns	0.7778	ns	0.6507	ns	0.9920

[1] The four combinations of oxygen exposure regimes during pressing and handling: inert-reductive (IR), inert-oxidative (IO), aerobic-reductive (AR), and aerobic-oxidative (AO).

Table S6a. Summary of the significant effects of oxygen exposure during pressing and handling on the metal ion concentrations in juice and wine lees.

	Aluminium		Calcium		Cobalt		Chromium		Copper		Iron	
**Sidak's multiple comparisons test**	**Adjusted P Value**		**Adjusted P Value**		**Adjusted P Value**		**Adjusted P Value**		**Adjusted P Value**		**Adjusted P Value**	
**Press conditions: Inert - Aerobic**												
Juice Lees	ns	0.9185	ns	>0.9999	ns	0.9242	ns	0.7287	ns	0.0596	ns	0.5507
Wine Lees	ns	0.9999	ns	0.4490	ns	0.9407	ns	0.9907	****	<0.0001	ns	0.7362
**Handling conditions: Reductive - Oxidative**												
Juice Lees	ns	0.7047	ns	0.9870	ns	0.9721	ns	0.6276	ns	0.8051	ns	0.7122
Wine Lees	**	0.0061	ns	0.3014	ns	0.1852	ns	0.9957	ns	0.8831	ns	0.3099

Table S6b. Summary of the significant effects of oxygen exposure during pressing and handling on the metal ion concentrations in juice and wine lees.

	Potassium		Magnesium		Manganese		Sodium		Nickel		Zinc	
**Sidak's multiple comparisons test**	**Adjusted P Value**		**Adjusted P Value**		**Adjusted P Value**		**Adjusted P Value**		**Adjusted P Value**		**Adjusted P Value**	
**Press conditions: Inert - Aerobic**												
Juice Lees	ns	0.8555	ns	0.9575	ns	0.9824	ns	0.7505	ns	>0.9999	ns	0.9940
Wine Lees	ns	0.4033	ns	0.6465	ns	0.9114	ns	0.8500	ns	0.9937	**	0.0064
**Handling conditions: Reductive - Oxidative**												
Juice Lees	ns	0.9991	ns	0.2817	ns	0.1073	ns	0.3120	ns	>0.9999	ns	0.8196
Wine Lees	ns	0.6263	ns	0.6053	ns	0.8614	ns	0.1579	*	0.0120	ns	0.9763
